# Recent Advancements in Nanomedicine for ‘Cold’ Tumor Immunotherapy

**DOI:** 10.1007/s40820-021-00622-6

**Published:** 2021-03-16

**Authors:** Qinjun Chen, Tao Sun, Chen Jiang

**Affiliations:** Key Laboratory of Smart Drug Delivery (Ministry of Education), State Key Laboratory of Medical Neurobiology and MOE Frontiers Center for Brain Science, Institutes of Brain Science, Department of Pharmaceutics, and School of Pharmacy, Research Center on Aging and Medicine, Fudan University, Shanghai, 201203 People’s Republic of China

**Keywords:** Tumor immune microenvironment, Cold tumor, Nanotechnology, Immunosuppressive, Combination therapy

## Abstract

Although current anticancer immunotherapies using immune checkpoint inhibitors (ICIs) have been reported with a high clinical success rate, numerous patients still bear ‘cold’ tumors with insufficient T cell infiltration and low immunogenicity, responding poorly to ICI therapy. Considering the advancements in precision medicine, in-depth mechanism studies on the tumor immune microenvironment (TIME) among cold tumors are required to improve the treatment for these patients. Nanomedicine has emerged as a promising drug delivery system in anticancer immunotherapy, activates immune function, modulates the TIME, and has been applied in combination with other anticancer therapeutic strategies. This review initially summarizes the mechanisms underlying immunosuppressive TIME in cold tumors and addresses the recent advancements in nanotechnology for cold TIME reversal-based therapies, as well as a brief talk about the feasibility of clinical translation.

## Highlights

- Mechanisms underlying immunosuppressive tumor immune microenvironment (TIME) in ‘cold’ tumor are summarized.
- Recent nanotechnology-based strategies for ‘cold’ TIME firing up are emphasized.
- Challenges and perspectives of nanomedicines for ‘cold’ tumor treatment are proposed.

## Graphical abstract

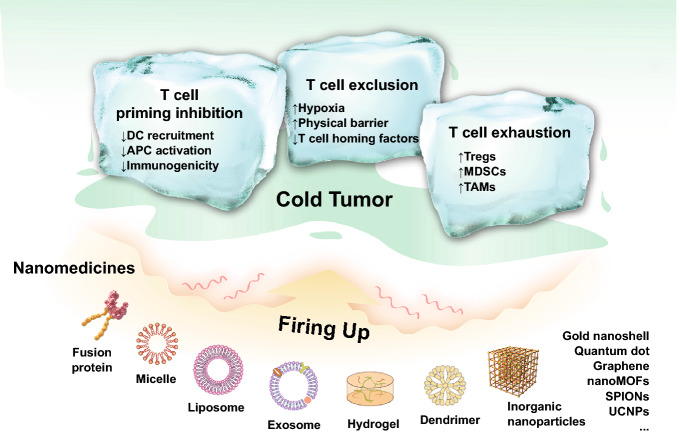

## Introduction

Immunotherapy has emerged as a novel and effective treatment for oncology patients by activating the host immune system to eliminate cancer cells [1]. Particularly, therapies involving immune checkpoint inhibitors (ICIs) have shown remarkable and long-lasting clinical outcomes in some advanced carcinoma, represented by programmed death protein-1 (PD-1) or its ligand (PD-L1) antibody and cytotoxic T-lymphocyte-associated antigen-4 (CTLA-4) antibody therapies. However, along with further clinical research, it was reported that the efficacy of ICI therapy was not uniform among cancer patients or cancer types, where only about 20% patients exhibited a positive T cell response and clinically benefited from this therapy [2–9]. Patients with favorable responses to ICI therapy always have a high level of tumor-infiltrating lymphocytes (TILs) in tumor lesions, and such tumors are normally labeled as hot tumors [10–12]. In contrast, patients responding poorly usually bear tumors with insufficient T cell infiltration, which are regarded as cold tumors [13]. In-depth mechanism studies on tumor immune microenvironment (TIME) of hot tumors have reported that immunosuppressive factors in hot tumors are involved in a negative feedback loop driven by TILs, such as the up-regulation of PD-L1, CTLA-4, and IDO [3, 14], which are rarely found in TIL-lack cold tumors. Instead, there are low immunogenicity and T cell exclusion in cold tumors [15, 16]. Furthermore, numerous clinical studies have proposed that prognostic and clinical outcomes of immunotherapy greatly relied on the T cell infiltration rate in various tumors [17–21]. Therefore, it is critical and challenging to increase T cell infiltration for cold tumors.

In general, effective T cell recruitment requires an enhanced immune circle in tumor lesions. First, the patrolling dendritic cells (DCs) are recruited to tumor lesions through inflammatory chemokines (such as CCL4) produced from tumors [22, 23] in early tumorigenesis. Thereafter, endogenous adjuvants (including cytosolic DNA) released from dying tumor cells activate the CD103^+^ subset of DCs to recognize the targeted tumor cells (via DNA-cGAS-STING pathway), following by type I interferon (IFN) generation which in turn recruits DCs and promotes DC translocation into tumor-draining lymph nodes (TDLNs) for the tumor-specific T cell activation [24–27]. At the TDLN stage, the maturity and functionality of DCs and immunogenicity of its presented epitopes determines the extent of T cell immune response [28, 29]. Then, the activated effector T cells enter the bloodstream and screen for inflammatory microenvironments through surface-expressed homing molecules, *i.e.,* P-selectin and E-selectin ligands and the chemokine receptor CXCR3 [30, 31]. Finally, accompanied with the selectin-mediated endothelial adhesion and chemokine-mediated integrin activation, effector T cells can thus successfully migrate into tumor tissues and kill tumor cells [32].

**Fig. 1:**
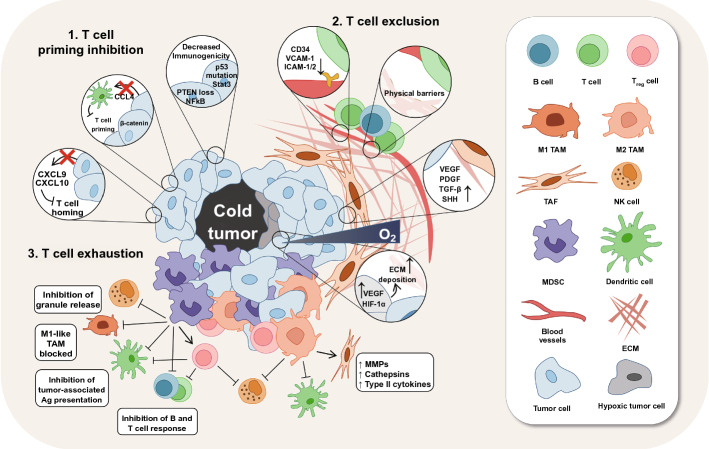
Immunosuppressive mechanisms of the tumor immune microenvironment in cold tumors. STAT3, signal transducers and activators of transduction-3; NF-κB, nuclear factor kappa-B; CCL4, CC-chemokine ligand 4; CXCL9/CXCL10, CXC-chemokine ligand 9/10; VCAM-1, vascular cell adhesion molecule 1; ICAM-1/2, intercellular adhesion molecule-1/2; VEGF, vascular endothelial growth factor; PDGF, platelet-derived growth factor; TGF-β, transforming growth factor-β; SHH, sonic hedgehog; ECM, extracellular matrix; HIF-1α, hypoxia-inducible factor 1α; TAM, tumor-associated macrophages; MMPs, matrix metalloproteinases; TAF, tumor-associated fibroblast; NK cell, natural killer cell; MDSCs, myeloid- derived suppressor cells

In the TIME of cold tumors, as expected, the immune circle is interrupted in three major pathways (Fig. 1). First is T cell priming inhibition, including decreased immunogenicity and failed antigen-presenting cells (APCs) and T cell recruitment, which is mainly caused by genetic mutation. Second is T cell exclusion. The deposition of extracellular matrix and stiff stroma-induced hypoxia in cold tumor lesions can build a physical and chemical barrier to obstruct the T cell infiltration. Furthermore, numerous immunosuppressive cells, represented by myeloid-derived suppressor cells (MDSCs), Regulatory T cells (Tregs) and tumor-associated macrophages (TAMs), are widely settled in the TIME of cold tumors and suppress the cytotoxicity of CD8^+^ T cells via T cell exhaustion. Therefore, a multiple combinatorial therapy addressing these characteristics of cold TIME is desiderated for reversing immunosuppressive TIME and conquering the cold tumors.

Nanomedicine is the medical use of nanoscaled cargo to prolong circulation time, to protect the loaded-drug from degradation, and to promote the accumulation and drug release into targeted tissues and cells, which has been extensively developed as diagnostic, therapeutic and preventive medicine in healthcare [33–38]. Up to date, there has been over 200 products of nanomedicines either approved or under clinical investigation, covering almost all types of nanomaterials, for example, liposomes (Doxil), protein-based nanoparticles (Abraxane), polymeric micelles (Apealea), liposomal gene delivery formulation (NCT02369198) and inorganic nanoparticles (Sienna^+^) [39]. Similarly, the application of nanomedicines in anti-tumor immunotherapy has also received enormous attention and shown great advantages over traditional strategies. Abraxane and Doxil are the two most studied products of nanomedicine in combination with immunotherapy, particularly with PD-1/PD-L1 antibody therapy, among which, the combination of Abraxane and atezolizumab has been the first immunotherapeutic regimen approved by FDA for locally advanced or metastatic triple-negative breast cancer [40]. Furthermore, autoimmune toxicity caused by nonspecific immune stimulation remains as a major challenge to current immunotherapies, and numerous studies have shown that delivery of immunomodulatory agents via nanocarriers can not only protect the cargo from leakage and degradation, but also enables its targeted accumulation, resulting in alleviated toxicity and reinforced T cell responses as opposed to systemic administration of free agents [41–43]. As expected, several advanced nanomedicines involving nucleic acids and vaccines have been processed in clinical trials, such as mRNA nanovaccine (lipo-MERIT) for melanoma (NCT02410733), anti-EGFR bispecific antibody minicells with microRNA for mesothelioma and non-small cell lung cancer (NCT02369198) and autologous cell vaccines for breast cancer (NCT00317603) [44].

As to cold tumors, co-stimulating multiple immunosuppressive pathways synergistically is on demand for APC- or T cell-based immune initiation [45–47]. However, it is difficult to accomplish this goal by systemic administration of various immunomodulatory agents due to their differences in the pharmacodynamic and pharmacokinetic properties. In this regard, incorporation of these agents in one nanoparticle would serve as an ideal approach for synergic drug exposure [44]. Furthermore, desmoplastic extracellular matrix (ECM) in cold tumor lesions can act as a physical barrier to both obstruct T cell infiltration and impair the permeation of free drugs, requiring the early breaching of the matrix barrier before the therapeutic process. It has been widely reported that various nanomaterials can endow the corresponding cargos with capability to penetrate the matrix barriers by particle size reduction or charge reversal from negative to positive upon meeting the enzymes, light or lower pH. Moreover, the highly modifiable chemical groups on the surface of the nanoparticles provide the feasibility to target multiple cells in the tumor microenvironment [17, 48–52]. In this review, we summarize the mechanisms underlying immunosuppressive TIME in cold tumors and address recent advancements in nanomedicine for cold TIME reversal-based therapies, as well as the feasibility of clinical translation of nanomedicine for immunotherapy.

## Application of Nanomedicines in Treating Cold Tumors

In recent years, as the rapid development of medical test and diagnostic procedures, there has been an increasing number of voices calling for treating cancer patients with precision medicine based on the specificity of TIME characteristics [53]. Meanwhile, various nanomaterials have been developed in accordance with the healthcare requirements. Therefore, in the following sections, the application of nanomedicines would be introduced according to the characteristics of TIME in cold tumor rather than material-dependence, which would be divided into three main categories, including strategies for T cell priming resumption, T cell exclusion overcoming and T cell exhaustion reversion. In each category, the characteristic of TIME would be outlined first, and the corresponding strategies would be proposed therewith.

### Strategies for T Cell Priming Resumption

#### Characteristics of T Cell Priming Inhibition in Cold Tumors

APCs (mostly DCs) play an important role in de novo generation of T cell specific immunity; however, APC functions are always disrupted by numerous factors in cold tumors [22, 54]. The Wnt-β-catenin pathway is the first identified tumor-intrinsic oncogene pathway mediating the disruption of APC recruitment in patients with cold tumor. During the activation of the Wnt-β-catenin signaling pathway, tumors induce activating transcription factor 3 (ATF3)-dependent transcriptional suppression of CCL4, a DC recruitment-supporting chemokine, thus reducing DC recruitment and inhibiting T cell priming [22, 55]. Meanwhile, activation of the Cox1/2-prostaglandin E2 (PGE2) pathway deters DC infiltration through the down-regulation of DC chemo-attractants CCL5 and XCR1 owing to natural killer (NK) cell impairment [56].

Furthermore, cytokines including macrophage colony-stimulating factor (M-CSF) [57], transforming growth factor β (TGF-β) [58], interleukin (IL)-6 [59], and IL-10 [60] in the TME potentially disrupt DC maturation and antigen presentation in tumors, thus inhibiting T cell priming. Meanwhile, MYC-driven up-regulation of CD47 [61], an antiphagocytic protein inhibiting the phagocytic effects of macrophages and DCs on tumor cells, on tumor cells can ultimately impair the potential of APCs to prime effector T cells.

Thirdly, alterations in the conformation and number of epitopes presented to APCs are the major mechanisms regulating antigen processing and presentation in APCs [62]. For example, the alterations in the proteasomal or post-proteasomal machinery can impair antigen processing by disrupting the epitope binding to major histocompatibility complex (MHC) molecules [63, 64], and mutations and epigenetic changes in MHC-I can regulate the presentation of processed epitopes on the tumor cell surface [65, 66]. The loss-of function mutations in PTEN or activation mutations in PI3K can reduce the autophagy in cold tumors, which results in decreased presentation of danger signals to APCs. Furthermore, antigenic discontinuum owing to the oncogenic mutations (including those in *KRAS*) or chromosome rearrangements (such as *BCR-ABL1*) can induce a non-destructive immune response that is aberrantly considered as tumor immunity [67–69].

#### DC Recruitment and Functional Enhancement

**Fig. 2:**
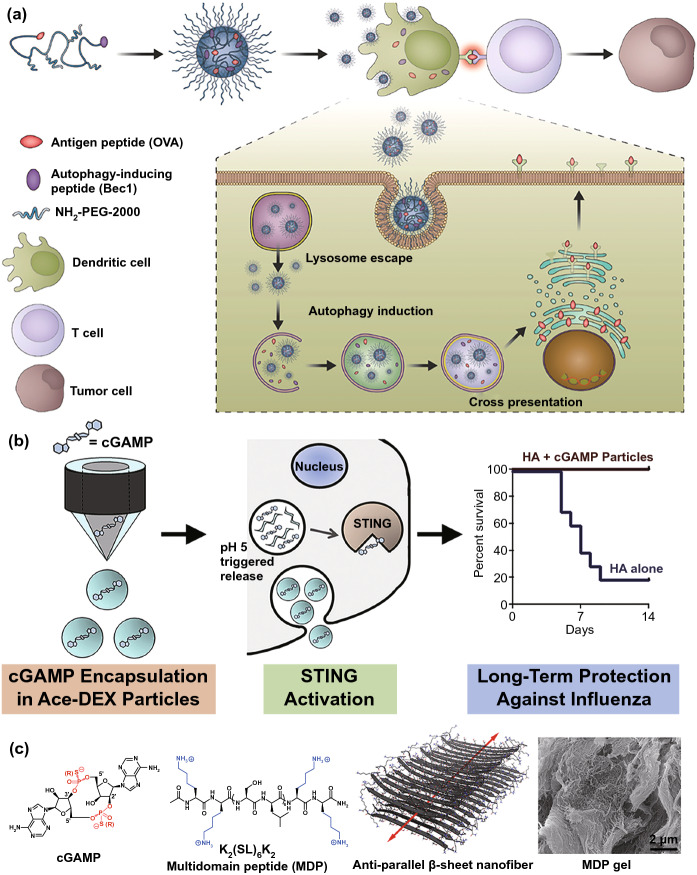
Nanomedicines for cGAMP. **a** In vivo up-regulation of the autophagy pathway in DCs via a nano-activator comprising the antigenic peptide and autophagy-inducing peptide (Bec1). Reproduced with permission from Ref. [77]. **b** pH-sensitive acetylated dextran (Ace-DEX) polymeric microparticles. Reproduced with permission from Ref. [73]. **c** Anti-parallel β-sheet nanofibrous hydrogels. Reproduced with permission from Ref. [78]

According to the characteristic of T cell priming inhibition, DC recruitment is the first step for T cell activation. CCL4 is a crucial chemokine for CD103^+^ DC recruitment; however, it is usually lacking in tumor lesions [70], and the retention of external CCL4 in tumor lesions is still a problem. Recently, a fusion protein of CCL4 and collagen-binding domain (CBD) of von Willebrand factor was generated to achieve stromal-targeted delivery of CCL4 to increase the retention of CCL4. As expected, intravenous administration of CBD-CCL4 can remarkably enhance the recruitment of CD103^+ ^DCs and CD8^+^ T cells, exhibiting significant anti-tumor effects in multiple tumor models in combination with ICB immunotherapy [26]. Furthermore, recent studies have revealed that autophagy in DCs can promote both MHC class I and II presentation of endogenous or exogenous antigens. Hence, Wang et al*.* tried to conjugate both autophagy-inducing peptide (Bec1) and OVA peptide to the terminals of a pH-responsive polymer to form a nano-activator for T cells activation [71] (Fig. 2a). In this work, they reported that the buffering capability and low-pH-triggered morphology swollen of nano-activator were beneficial to endosomal escape and peptide exposure, which could increase the cross talk between autophagy and functional antigen presentation, eventually leading to the high-efficiency antigen cross-presentation and antigen-specific T cell generation. Stimulator of IFN genes (STING) plays a crucial role in cyclic dinucleotide (CDN)-driven DC maturation [72], and its potent agonist, 2′3′-cyclic guanosine monophosphate-adenosine monophosphate (cGAMP), are widely used to activate DCs in encapsulation with various cationic nanocarriers including cationic liposomes [73]. Nevertheless, the toxicity of these macromolecular cationic materials and inefficient cytosolic cargo transport greatly limit the cGAMP delivery [74, 75]. Currently, some novel pH-sensitive cationic polymers offer the potential solutions. A pH-responsive cross-linkable polymersome was developed by Shae et al*.* to encapsulate cGAMP to promote endosomal escape of cargos in response to endolysosomal acidification to disassemble membrane-destabilizing segments [76]. In another study, cGAMP was encapsulated into pH-sensitive acetylated dextran (Ace-DEX) polymeric microparticles (MPs) to achieve an approximately 50-fold in vivo increase in type I IFN responses I comparison with soluble cGAMP [77] (Fig. 2b). Furthermore, a previous study introduced thixotropic and extracellular matrix-mimicking multi-domain peptides (MDPs) to load cGAMP in the form of anti-parallel β-sheet nanofibrous hydrogels in solution, which markedly improved the overall survival in a challenging murine model with an eightfold lower cGAMP release rate as opposed to standard collagen hydrogel-mediated delivery [78] (Fig. 2c).

**Fig. 3:**
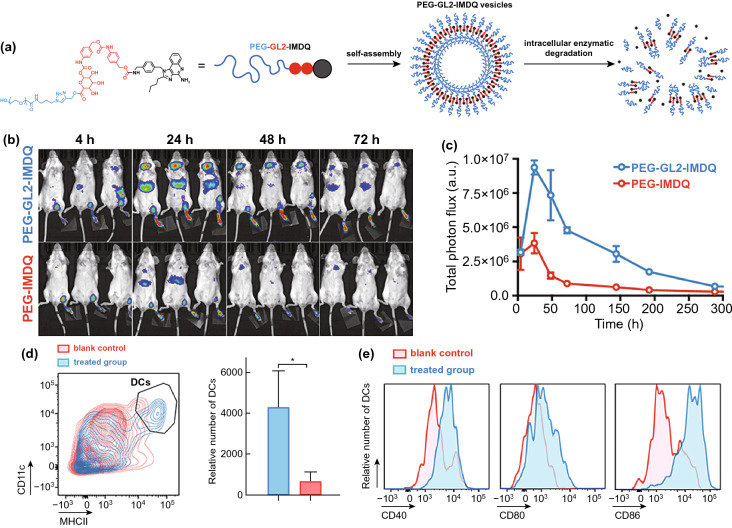
**a** Schematic representation of the fabrication of IMDQnano: block copolymers composed of mTEGMA and PFPMA self-assemble in DMSO into micellar nanoparticles driven by solvophobic interaction between PFPMA moieties. Covalent IMDQ-ligation, cross-linking with a pH-sensitive cross-linker, and transformation of unreacted PFP esters into hydrophilic repeating units yield stable hydrogel nanoparticles that can disassemble into soluble polymers in response to an acidic pH. **b** Size distribution measured by DLS and IMDQ loading of IMDQnano and empty nanoparticles. **c** TEM images of IMDQnano and empty nanoparticles. **d** Tumor growth of B16 tumors in mice, in response to treatment monotherapy and combination therapy (n = 6). **e** Confocal microscopy image of a tissue section collected from a B16 tumor treated with IMDQnano, anti-PDL1, and Flt3L combination immunotherapy. **f** Confocal microscopy image of a tissue section collected from a B16 tumor treated with PBS control. Reproduced with permission from Ref. [83]

Adjuvants, such as the agonists of Toll-like receptors (TLR), are a major class of immune-modulatory molecules that can effectively activate DC cells and promote antigen presentation to T cells [79]. Currently, there are 11 types of TLRs found in the cells and their locations that can be divided into two categories: on cytomembrane or endosomal membrane [80]. Particularly, TLR1, TLR2, TLR4, TLR5, TLR6, TLR10, and TLR11 are located on cytomembrane, and TLR3, TLR7, TLR8, and TLR9 are located on endosomal membrane. As for the delivery of TLR agonists, releasing the cargo in appropriate location can maximize the efficacy of agonists, while avoiding rapid diffusion of agonists from the site of injection is also the concerns we should take seriously so as to reduce the severe systemic inflammation. In regard to the cell membrane-located TLR activation, adequate and effective exposure of their agonists to cell surface is warranted. Monophosphoryl lipid A (MPLA) is an FDA-approved detoxified derivative of lipooligosaccharide (LOS) with immune stimulatory effects through engagement of TLR4. According to the TLR4 located on cell membrane, Traini et al*.* [81] developed a nanovaccine featuring MPLA adhered to mIONPsp through hydrophobic interactions and model antigen (OVA) linked by hydrazine bonds to create adjuvant-exposed and antigen-protected nanostructures for maximum TLR4-based DC activation, and the results indicated that it would be a promising strategy to improve the immunostimulatory properties and reduce cytotoxicity through exposed delivery of MPLA and OVA by mIONPsp. Furthermore, it’s also critical to release cargo from the nanovaccine in endosome for delivery of endosomal membrane-located TLRs’ agonists. Thus, the mildly acidic and hydrolase-rich microenvironment of endosome might be served as trigger for controlled drug release. For instance, Nuhn et al*.* [82] developed a self-assembled, pH-degradable TLR7/8 agonist-ligated nanoparticle carrier (IMDQnano) that can protect TLR7/8 agonist from systemic bio-distribution and unfavorable degradation, while retaining the valid anti-tumor efficacy of localized IMDQ treatment. Meanwhile, a study from Wang et al*.* [83] reported an amphiphilic conjugation of TLR7/8 agonist to poly (ethylene glycol) (PEG) via endosomal enzyme-responsive linker that self-assembled to form a nano-vesicular structure to achieve lymph node-focused drug delivery and enzyme-triggered release of native drugs after endocytosis, eventually inducing robust maturation of DC cells in vivo (Fig. 3).

Finally, artificial DC-derived nano-vaccines, regardless of DC membrane coating formulations [84] or DC-derived microvesicles [85], have been reported to have the inherent potential of antigen presentation and T cell stimulation from activated DCs and to exert marked therapeutic and prophylactic effects against tumors.

#### Synchronous Delivery of Adjuvants and Neoantigens

Efficient induction of antigen-specific adaptive immunity requires two conditions: successful tumor-specific neoantigen delivery to APCs and sufficient APC activation, a so-called adjuvant effect [86–88]. Recent studies on nanotechnology-based co-delivery strategies, apart from ICD induction [89], have reported that subunit antigens (including ovalbumin [90]) and antigen-coded mRNAs [91] are the most commonly used model antigens, and these applied adjuvants were primarily classified as TLR agonists including lipopolysaccharide (LPS) for TLR4 [92], imiquimod and resiquimod for TLR7/8 [93], and CpG for TLR9 [94].

**Fig. 4:**
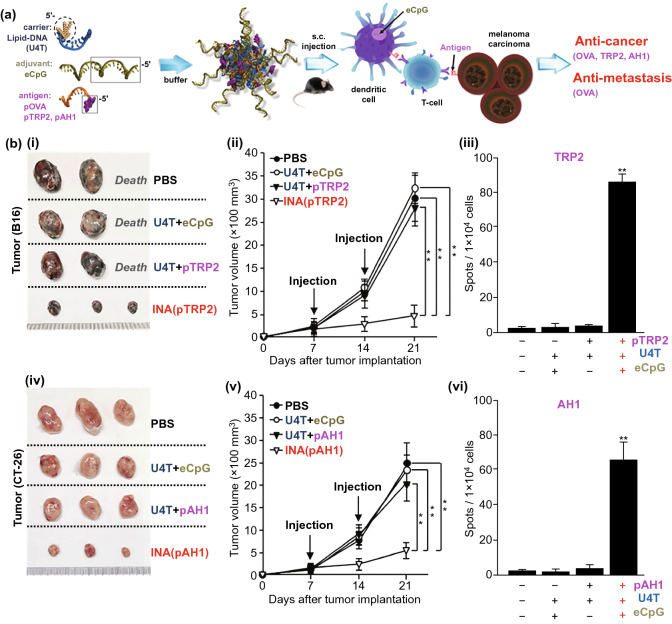
**a** Carrier (U4T) is a lipid-DNA that consists of four dodec-1-ynyluracil (U) nucleobases at the 5′-end and eight regular deoxyribonucleotides. In aqueous media, lipid-DNA strands are hybridized with eCpG. At the 5′-end, the 20-nucleobases long murine TLR-9 ligand (phosphorothioate linkage, italicized) was extended with 12-bases complimentary to the lipid-DNA and antigen (pOVA, pTRP2 or pAH1, Ovalbumin1 (OVA) peptide epitope (AA sequence: SIINFEKL, magenta) is covalently attached to a peptide nucleic acid sequence complementary to lipid-DNA (orange).) in an equimolar ratio. Thus, immunotherapeutic nucleic acid (INA) loaded with both multiple adjuvants and antigens is formed. Subcutaneous injection of INA into tumor-bearing mice induces an antigen-specific immune response via CD8α + DCs and subsequent presentation of antigen via regulatory T cells. The antigen-specific immune stimulation by INA results in successful therapeutic effects (inhibited growth of multiple tumors and metastasis). **b** Cancer Ag-specific anticancer effect of INA, including cancer Ag of melanoma (TRP2) or carcinoma (AH1). (i) Sizes of tumor masses on day 21 after introduction of the melanoma tumor cells, (ii) B16 melanoma tumor growth over time, (iii) TRP2 peptide-specific IFN-γ production was measured in tumor-drLN by ELISpot. Mean ± SEM (n = 6). **p < 0.01. (iv-v) BALB/c mice were injected s.c. with CT-26 carcinoma cells and treated with INA on day 7 and 14 following tumor injection, iv) sizes of carcinoma tumor masses on day 21, (v) tumor growth curve of carcinoma, vi) AH1 peptide-specific IFN-γ production and the mean number of spots are shown. Mean ± SEM (n = 6). **p < 0.01. Reproduced with permission from Ref. [95]

Simultaneous co-delivery of specific antigen epitopes and immunostimulant moieties is required for efficient vaccine immunogenicity [45]. Owing to the nucleic acid-like property of CpG and the facile modification of nucleic acids, Jin et al. [95] reported a self-assembling lipid-DNA-peptide nano-aggregation (INA) hybridized with the CpG motif, lipid-DNA, and antigen peptide-DNA for co-delivery of multiple adjuvants and antigens together (Fig. 4). The hybrid-DNA nanostructures could efficiently co-deliver the antigen peptide and CpG to CD8α^+^ DCs in the tumor-DLN, promoting potent antigen presentation to regulatory T cells via DC activation. Finally, INA treatment could exhibit anti-tumor and anti-metastasis effects against carcinoma and melanoma in vivo. Another study reported a generalizable conjugation approach for co-delivering peptide antigens and adjuvants in a self-assembling vaccine platform (SNP-7/8a) of uniform size (~ 20 nm) [96], containing hydrophilic positive charge-modified antigen peptide, cathepsin degradable linkers, and hydrophobic poly-TLR-7/8 agonists. Subcutaneous vaccination of SNP-7/8a containing 179 types of predicted neoantigens in mice activated CTLs against ~ 50% of neoantigens with high predicted binding affinity of MHC-I, thus reinforcing tumor clearance.

#### Targeted Intracellular Delivery of Adjuvants and Neoantigens

Endogenous antigen can directly activate antigen-specific CTLs via MHC-I-mediated antigen presentation, while exogenous antigens are presented by MHC-II to activate regulatory T cells, which facilitates CTL-mediated cellular immunity or B cell-mediated humoral immunity [97]. Hence, cytosolic delivery of exogenous antigens is necessary for the antigen to be considered an endogenous antigen and presented to CTLs via MHC-I. However, most adjuvants are ligands of TLR7/8/9, expressed in DC endosomes [98]. In order to deliver not only adjuvants, such as CpG-DNA, to endosomes but also antigens to the cytosol of DCs, Yoshizaki et al*.* developed liposomes modified with a pH-sensitive polymer (MGlu-HPG) loaded with cationic lipids (TRX), Toll-like receptor 9 ligand (CpG-DNA), and the antigen peptide (ovalbumin) for efficient antigen delivery and DC activation [94]. After the classic receptor-mediated endocytosis, adjuvants, TRX, and CpG-DNA bound to their cognate endosomal receptors and stimulated DC activation (up-regulation of co-stimulatory molecules and cytokine production). Thereafter, owing to the pH-sensitive membrane fusion potential of MGlu-HPG, the endosomal membrane was gradually degraded and the loaded antigen was released into the cytosol for MHC-I-mediated cross-presentation to CTLs, resulting in high immunity-inducing effects for effective cancer immunotherapy.

#### mRNA-Based Vaccines

Recently, mRNA encoding antigen-based vaccines have received increasing attention for developing anti-tumor immunity [99, 100]. Compared with conventional live-attenuated or subunit vaccines, the advantages of mRNA-based vaccines are obvious [101], including less safety concerns, the potential to produce various personalized tumor antigens with GMP quality, and intra-cytosolic translation of selected antigens in DCs, which can be directly presented to CTLs via MHC-I [97]. Nano-delivery strategies have facilitated effective intra-cytosolic delivery of mRNAs in DCs; however, it is not that easy to successfully translate the delivered mRNAs into coding antigens in DC cells. Verbeke et al*.* and other groups have reported that foreign mRNA is usually recognized by the intracellular danger-sensing receptors, such as TLRs, thus inducing an innate immune response and burst release of type I IFNs, which in turn reduce the mRNA stability and further translation [91, 102, 103]. Therefore, it is critical to develop a delivery system that can not only protect mRNA, but also enable efficient release and translation at the appropriate time for mRNA vaccine delivery. Primarily, researchers have generated nucleotide-modified mRNA to protect mRNA by preventing the release of mRNA recognition-associated type I IFNs; however, this strategy resulted in the loss of mRNA-related self-adjuvant effects, thus affecting DC activation and T cell priming. Furthermore, they developed a lipid nanoparticle encapsulating nucleoside-modified mRNA and TLR4 agonist monophosphoryl lipid A (MPLA) to ensure both antigen up-regulation and moderate the DC activation [91]. Similarly, to compensate for reduced DC stimulation owing to the reduced type I IFN, they assessed a combined delivery tactic of immune-silent nucleoside-modified mRNA and activator of invariant natural killer T cells (iNKT), glycolipid α-galactosylceramide (α-GC) in their recent study [104]. In this study, α-GC served as an indirect adjuvant for inducing controllable DC activation via bidirectional activation between iNKT cells and α-GC-presenting DCs, thus contributing to sevenfold tumor infiltration of antigen-specific CTLs than the current “gold standard” on the administration of mRNA vaccines.

#### Lymph Node Targeting

**Fig. 5:**
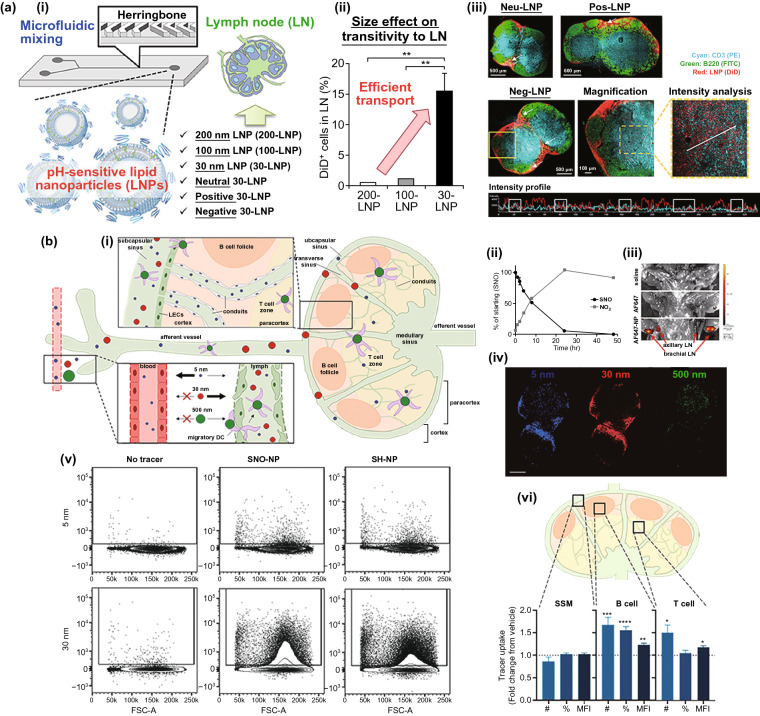
**a** (i–ii) Scheme of effect of size and charge of lipid nanoparticles prepared by microfluidic mixing on their lymph node transitivity and distribution. (iii) CLSM images of LNs treated with each LNP. The white arrows represent yellow dots showing the colocalization of B220^+^ cells and LNPs. An intensity analysis was performed in the range of the white line. White squares in the intensity profile represent the colocalization of CD3^+^ cells and LNPs. Cyan, green, and red show CD3 (PE), B220 (FITC), and LNP (DiD), respectively. Reproduced with permission from Ref. [108]. **b** (i) Schematic of size-based effects on molecule drainage from the interstitium and into draining LNs (dLNs). Blue = 5 nm, red = 30 nm, and green = 500 nm molecules, (ii) S-nitrosothiol (SNO) and nitrite (NO_2_) concentration in a solution of SNO-NP over time, (iii) IVIS imaging of AF647-labeled NP draining to axillary and brachial LN from a forelimb injection, (iv) confocal microscopy images of tracer distribution within a brachial LN 72 h after ipsilateral forelimb injection. Scale bar = 500 μm, (v) representative examples of CD45 + LN cells without 5 and 30 nm tracers, or with tracer and SNO-NP or SH-NP treatment, vi) effect of SNO-NP treatment on tracer uptake by barrier, cortex, and paracortex cell populations. Reproduced with permission from Ref. [113]

Lymph nodes (LN) are crucial secondary lymphoid organs where abundant APCs and T cells reside and interact for immune surveillance and responses against pathogens and tumors [105]. Therefore, increasing the transport of vaccines to LNs would be a beneficial strategy for reinforcing the immune responses. Numerous previous studies have demonstrated that nano-vaccines containing antigens and adjuvants exhibited the superior capability of increasing lymph node accumulation due to their cargo protection and lymph node-targeting transport [106]. In general, there are two ways for peripherally administrated vaccines traveling to lymph nodes that is passive transport through afferent lymphatics or peripheral DC cells-mediated active transport. Nowadays, it is a common agreement that translocating vaccines through the passive transport is much more efficient than the active pathway [107]. Thus, we will focus on the novel strategies that reinforced the lymph node accumulation via passive transport in this section. Recently, Nakamura et al*.* [108] reconfirmed that the negatively charged 30-nm-sized lipid nanoparticles (LNPs) were more efficiently translocated to the deep cortex of LNs and taken up by CD8^+^ DC cells through afferent lymphatics than the larger-sized LNPs or neutral/positively charged 30-nm-sized LNPs (Fig. 5a). Many researchers [106] also suggested that modifying the small-sized nanoparticle with DC-targeting molecular (such as, mannose [109], Fc receptor [110] or alginate [111]) could improve the possibility of nanoparticles being ingested by DC cells after entering the lymphatic vessels, thereby facilitating their trafficking to LNs. Similarly, endogenous vectors, represented by albumin, with the capability of entering lymphatics in the interstitium can also be exploited for LN-targeting drug delivery. Irvine group [112] was the pioneer to discover that long-chain fatty acids or hydrophobic molecules can bind to albumin with hydrophobic interaction, and they have developed a variety of long-chain fatty acids coupled adjuvant products and demonstrated their readily facilitating LN accumulation of adjuvants by hitch-hiking the albumin. Furthermore, several strategies focusing on the regulation of lymphatic vasculature have also been developed to facilitate nanoparticle LN delivery. Nitric oxide (NO) is a potent agonist for vascular and lymphatic vessel dilation. Recently, Sestito et al*.* [113] employed a controlled NO release nanocarrier (SNO-NP) to investigate the effect of lymphatic-targeted NO on LN accumulation, distribution and uptake of co-delivered nanoparticles. In this study, they demonstrated that a sustained NO release in the lymphatic system could expand the lymphatic vessel and enhance the LN penetration, as well as the lymphocytic uptake of co-delivered nanoparticles after *i.d.* injection (Fig. 5b). Besides, Park et al*.* [114] reported that pre-treatment with chitosan can improve the movement of nanoparticles across the vaginal epithelium to LNs.

#### ICD Induction

The heterogeneity of tumor antigens and inevitable systemic safety concerns of adjuvants are two major challenges associated with the development of an effective co-delivery system for adjuvants and antigens to induce antigen-specific CTLs [115–119]. Ideally, each patient requires personalized treatment. Recent treatments stimulating tumor cells to undergo immunogenic apoptosis and facilitating in situ exposure of multiple neoantigens and DAMPs, termed as immunogenic cell death (ICD), have received increasing attention in personalized medicine [89, 120–122]. Apart from neoantigen release, the major immunogenic characteristics of ICD are mediated through DAMP exposure, including surface exposure of calreticulin (CRT), ATP secretion, and post-apoptotic exodus of heat shock proteins (HSPs) and high mobility group protein B1 (HMGB1). In particular, secreted ATP stimulates the intra-tumoral recruitment of APCs and CTLs, surface-translocated CRT serves as an ‘eat me’ signal for DC phagocytosis, and milieu-released HSPs and HMGB1 serve as activators for DC maturation and promote antigen presentation to CTLs. Ultimately, ICD treatment results in a vaccine-like effect in situ, leading to an antigen-specific immune response.

Certain current chemotherapeutics [123–125] including doxorubicin, mitoxantrone, oxaliplatin, and cyclophosphamide, radiotherapy (RT) [126], photodynamic therapy (PDT) [127], photothermal therapy (PTT) [128], and magnetic hyperthermia (MT) [129] are frequently reported ICD inducers. ICD refers to apoptosis due to ROS [130], and current studies on ICD induction are aimed at enhancing the ROS-inducing effect of ICD inducers through reformation or combination strategies [131–134]. For most chemotherapeutic ICD inducers, stimulating ICD-associated danger signaling is usually a collateral effect of their cytotoxicity [135]. Thus, we developed a combinatorial treatment strategy involving oxaliplatin and ferroptosis as prodrug-loaded Fe_3_O_4_ nanoparticles, to improve the ICD effect of oxaliplatin [132]. With advancements in biomedical equipment, micro-invasive PTT and PDT have received increasing attention owing to their accuracy and high efficiency during tumor destruction and during ROS-associated ICD induction [136–139]. For example, Zhang et al*.* [140] proposed that the tumor starvation therapy performed by PTT-inducing gelation shrinkage could comprehensively suppress the tumor growth, whether the tumor is in situ, metastatic or recurrence.

Furthermore, Zhou et al*.* reported combination of PDT and chemical ICD inducer via TME-stimulating prodrug-loaded vesicle could synergistically reinforce the growth inhibition of both primary and abscopal tumors in addition to CD47 blockade [52]. Meanwhile, Chen et al*.* put forward that upon irradiation, mitochondrial targeted aggregation-based emission photosensitizers [131] could evoke superior and larger-scale ICD than the popularly used photosensitizers, such as pheophorbide A and chlorin e6. Moreover, apart from encapsulating PSs into organic nanocapsules or attaching PSs onto nanoparticle surfaces, the metal–organic framework (MOF) self-assembled from PSs and metal clusters by coordination bonds have displayed great potential as nano-PSs for PDT within a high PS loading but less self-quenching [137, 141–143]. Besides, Park et al*.* reported that the 90-nm-sized nanoMOF exhibited 1.7-fold PDT efficacy than free PS [144] and Shao et al*.* developed a core–shell heterostructure comprising a UCNP core and porphyrinic an MOF shell for enhanced anti-tumor activity of combined PDT, hypoxia-activated chemotherapy, and immunotherapy [145, 146]. Meanwhile, some studies introduced low‐dose deeply penetrating X‐ray as alternatives, with the incorporation of high‐Z elements as transducers, thus enabling radiodynamic therapy to significantly cause tumor regression at very low X-ray doses with less side effects [147–149].

**Fig. 6:**
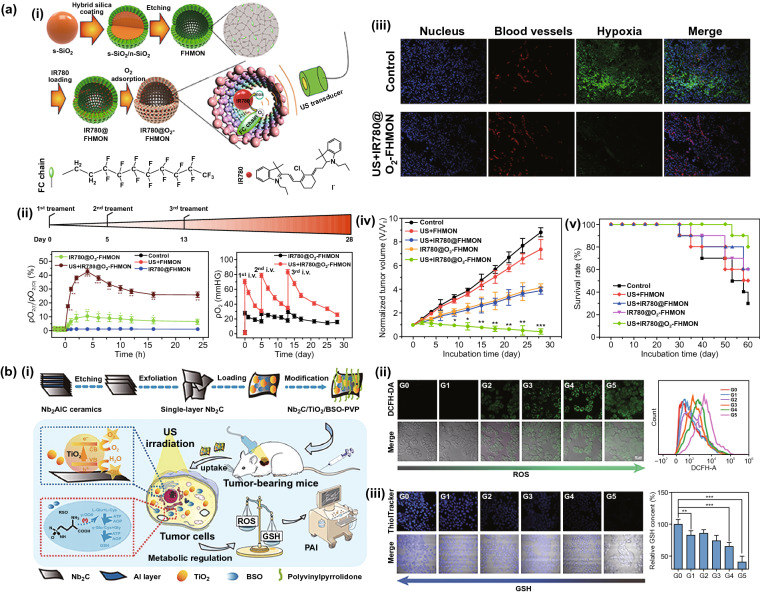
**a** (i) Synthetic process and action principle of IR780@O_2_-FHMON and characterization of FHMON carriers, (ii) In vivo therapeutic scheme of SDT on mice tumor xenograft and Relative pO_2_ variation of PANC-1 solid tumor after the first treatment within 24 h, significance was obtained via comparing to control (*, **, and *** represent p < 0.01, 0.005, and 0.001, respectively), as well as pO_2_ of PANC-1 solid tumor during the complete SDT experimental period, (iii) LCSM images of nuclei, blood vessels, and hypoxic regions stained by DAPI, hypoxia probe, and CD31 immunochemical methods in PANC-1 solid tumor slices of all groups at day = 28, (iv) time-dependent tumor volume variation of PANC-1 solid tumor treated with the above different groups. Data are presented as the mean value ± SD (n = 6), significance is obtained via comparing to the control group (*p < 0.01, **p < 0.005, and ***p < 0.001), (v) survival rate of tumor-bearing nude mice after treatments with the above different groups during the complete experimental period. Reproduced with permission from Ref. [151]. **b** (i) Schematic on the preparation process and enhanced SDT mechanism of Nb_2_C/TiO_2_/BSO-PVP, (ii) CLSM images and corresponding FCM data of 4T1 cells after different corresponding treatments in G0-G5 and subsequent DCFH-DA staining, (iii) CLSM images of 4T1 cells after different corresponding treatments in G0-G5 and subsequent ThiolTracker Violet dye staining, scale bar = 50 nm, as well as relative intracellular GSH content in 4T1 cells determined by Ellman’s reagent after different corresponding treatments in G0-G5. Data are expressed as mean ± standard deviation (SD) (n = 3) and *p < 0.05 and ***p < 0.001, which were obtained using t-student test. Note: G0-G5 represent Control, Nb_2_C/TiO_2_/BSO-PVP, US, TiO_2_-PVP + US, Nb_2_C/TiO_2_-PVP + US, and Nb_2_C/TiO_2_/BSO-PVP + US, respectively. Reproduced with permission from Ref. [153]

Since the ultrasound is non-radiative, which is superior to laser and X-ray in terms of penetration, sonodynamictherapy (SDT) has attracted increasing interest in ICD induction. Yue et al*.* [150] proved that combination therapy of checkpoint blockade and SDT based on clinically approved material comprising HMME/R837@Lip can not only reduce the tumor growth, but also prevent metastases and re-challenged tumor in mice. However, the low ROS generation caused by poor energy conversion efficiency from ultrasound to ROS-related chemicals is a challenge limiting SDT adoption. In order to improve the outcome of SDT, Zhang et al*.* contributed greatly. As oxygen is the key substrates for ROS production, they fluorinated the hollow mesoporous organo-silica nanoparticles (HMONs) for oxygen immobilization, as well as narrowing the distance between sonosensitizer (IR780) and oxygen, eventually relieving hypoxic and facilitating the efficiency of SDT against PANC-1 solid tumor [151] (Fig. 6a). In addition, they have enabled SDT-based nanoparticles with continuous CO_2_ bubble generation to promote ROS production and enhance the effect of ICD against breast carcinoma [152]. Recently, there is a growing perspective that induced ROS can be depleted by reductive species (such as GSH) for intra-tumoral redox metabolism equilibrium. In this regard, Guan et al*.* [153] developed metabolism-engineered and SDT-based nanoplatform (Nb_2_C/TiO_2_/BSO-PVP) wherein GSH synthesis inhibitor and sonosensitizer (TiO_2_) are accommodated by the Nb_2_C nanosheets to reduce ROS depletion and so as to improve the ROS production synergistically (Fig. 6b).

### Strategies for Overcoming T cell Exclusion

#### Characteristics of T cell Exclusion in Cold Tumors

Binding of chemokine ligands CXCL9 and CXCL10 to CXCR3 is required for active T cell migration and infiltration into tumor beds [30]. However, this phenomenon is normally suppressed in cold tumors. Histone modifications and DNA methylation are the major epigenetic factors that can directly down-regulate *CXCL9* and *CXCL10*, resulting in decreased T cell recruitment [154, 155]. Furthermore, R-2-hydroxyglutarate generation associated with mutations in isocitrate dehydrogenase 1 and 2 (*IDH1* and *IDH2*, respectively) can inhibit intra-tumoral CXCL9 and CXCL10 production [156]. Since CXCL9 and CXCL10 are IFN-responsive genes [157], the factors affecting CD103^+^ DC recruitment, such as Wnt-β-catenin pathway activation, might decrease T cell infiltration by affecting type I IFN expression.

Furthermore, the activation of tumor-associated fibroblasts (TAFs) and subsequent extracellular matrix (ECM) deposition might be another factor affecting T cell infiltration at tumor sites by the formation of physical barriers [158, 159]. TAF heterogeneity is associated with their tissue of origin, e.g., TAFs are regarded to originate from the bone marrow-derived precursors (BMDP), mesenchymal stem cells (MSC), liver and pancreas stellate cells, resting tissue fibroblasts and probably from several certain types of epithelial cells [160]. First, tumor cells harboring KRAS or NF-κB mutations release mitogenic and fibrogenic factors that can reprogram normal pro-fibrotic cells into active TAFs, including vascular endothelial growth factor (VEGF), platelet-derived growth factor (PDGF), TGF-β, and sonic hedgehog (SHH) [161–164]. Thereafter, a positive feedback expression of pro-inflammatory cytokines (including VEGF and TGF-β) produced from TAFs further promotes the TAF activation and promotes the production of abundant ECM components (including collagens, glycoproteins, elastin, and hyaluronan), resulting in extensive ECM deposition. The dense and compact ECMs tend to serve as a physical barrier to the entry of oxygen and nutrients and an impediment to active T cell migration. Furthermore, the activated TAFs produce factors including CXCL12 to limit the T cell recruitment to tumor lesions [165].

Third, the growing desmoplastic stroma can obstruct inner tumor cells from blood vessels, thus generating a highly hypoxic tumor cell-rich islet. In turn, the hypoxia-induced positive feedback loops reinforce ECM deposition by up-regulating pro-inflammatory cytokines including VEGF and TGF-β, while this further deters the T cell infiltration [162]. Simultaneously, hypoxia microenvironment increases the demand for tumor angiogenesis through hypoxia-inducible factor-1α (HIF-1α)-mediated up-regulation of various growth factors (represented by VEGF) [166], which in turn activates the FasL on tumor endothelial cells, thus triggering apoptosis in T cells upon binding to Fas expressed on T cells and reducing T cell infiltration [167]. Owing to the up-regulation of pro-angiogenic signaling (*e.g.,* VEGF signaling), the tumor neovascularization network is weakened, with a lack of pericyte coverage and loose endothelial cell junctions, thus disrupting blood vessel integrity and affecting blood flow [168]. Such increased neovascular permeability facilitates the extravasation of plasma proteins including fibrin, facilitating the influx of fibroblasts and inducing ECM deposition [158]. Moreover, VEGF up-regulation can induce a clustering defect among leukocyte adhesion molecules on endothelial cells, which deters the T cell extravasation, *e.g.,* intracellular adhesion molecule-1/2 (ICAM-1/2), vascular cell adhesion protein-1 (VCAM-1), and CD34 [169].

#### Stroma Normalization

**Fig. 7:**
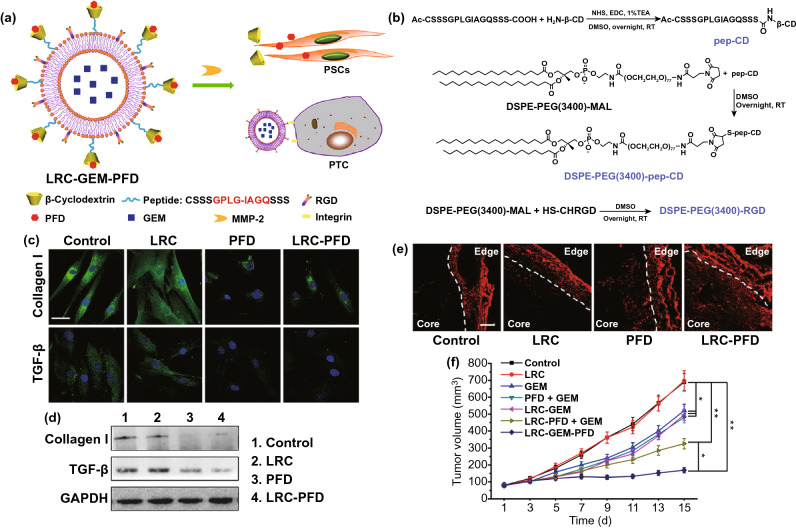
**a** The PFD inserts into the hydrophobic chamber of β-CD, and the GEM was encapsulated in the liposome. If the LRC-GEM-PFD is cleaved by MMP-2, the PFD part will regulate the PSCs, and the GEM containing liposome will recognize and kill the PTCs. **b** Synthesis of pep-CD, DSPE-PEG(3400)-pep-CD, and DSPE-PEG(3400)-RGD. **c** The immunofluorescence staining of collagen I and TGF-β. Blue: HOECHST, green: FITC-labeled collagen I or TGF-β. The scale bar: 50 μm. **d** The Western blot analysis of the collagen I and TGF-β in the conditioned medium of PSCs. **e** Penetration of Rhd into pancreatic tumor (Panc-1 and PSCs coimplanted) tissues after intravenous injection of different PFD formulations. Red: Rhd. The scale bar is 100 μm. **f** Tumor growth curves of PSCs/Panc-1 pancreatic tumors in mice treated by different GEM formulations. GEM dose: 20 mg/kg. Data were presented as mean ± standard deviation *p < 0.05, **p < 0.01 vs control. Reproduced with permission from Ref. [179]

Currently, three types of approaches for overcoming physical barriers have been reported: incentive control, matrix component degradation, and penetrating-nanoparticle exploitation. First, strategies for incentive control, also known as stroma normalization [170, 171], are primarily focused on inhibiting known factors contributing to matrix deposition, including VEGF [172], TGF-β [173], CXCL12 [174], HIF-α [175], and the hedgehog pathway [176]. Chen et al*.* reported that normalization of tumor vasculature upon being treated with EGFR inhibitors at a moderate dose could not only improve the tumor perfusion of oxygen and nanoscale therapeutic agents, but also alter the immunosuppressive TME by relieving the tumor hypoxia [170]. Another study reported that blockade of CXCR4/CXCL12 signaling with Plerixafor could also alleviate the desmoplasia and immunosuppression, in turn decompressing tumor vessels and increasing T cell infiltration, eventually enhancing immunotherapy in cold breast tumors [177]. Moreover, Papageorgis et al*.* reported that suppression of TGF-β signaling with antifibrotic drugs can significantly enhance the efficacy of nanoparticles with various sizes by reducing the ECM, decreasing interstitial fluid pressure, and improving tumor perfusion [178]. Furthermore, Ji et al*.* developed a β-cyclodextrin (β-CD)-modified matrix metalloproteinase-2 (MMP-2)-responsive liposome for co-delivery of antifibrotic and chemotherapeutic drugs [179] (Fig. 7). Upon the MMP-2 cleavage in the TME, the antifibrotic drug pirfenidone in β-CDs was maintained in the stroma and suppressed TGF-β and collagen I in pancreatic stellate cells (PSCs), thereby down-regulating fibrosis and decreasing the stromal barrier, thus enhancing drug-encapsulated liposome perfusion, ultimately improving the efficiency for pancreatic cancer therapy without overt side effects.

#### Matrix Degradation

**Fig. 8:**
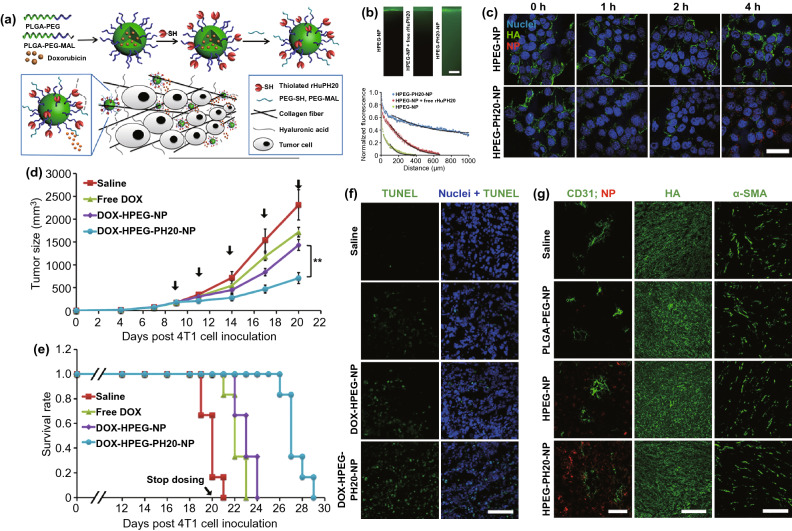
**a** Schematic illustration of NP fabrication and penetration in tumors via the degradation of hyaluronic acid (HA). The NPs were fabricated by conjugating thiolated rHuPH20 on the first PEG layer followed by anchoring the second PEG layer. **b** NP diffusion in ECM-mimicking gels. (Scale bar: 200 μm). The gels composed of 6.5 mg mL^−1^ of rat collagen I and 1 mg/mL of HA in capillary tubes. Ten microliters of 1 mg mL^−1^ of NPs (green) were added on the top of gels and incubated at 37 °C for 1.5 h before being imaged. The activity of free or conjugated rHuPH20 was 500 U mL^−1^ and normalized NP fluorescence with diffusion distance in gels. Images were analyzed via ImageJ. Diffusion coefficients were obtained by fitting the data to a one-dimensional diffusion model in MATLAB. Black lines display theoretical intensity profiles for particles with diffusion coefficients of 1.66 × 10^−7^, 7.17 × 10^−8^, and 1.11 × 10^−8^ cm^2^ s^−1^. **c** Confocal microscopy images of 4T1 cells treated with 0.02 mg mL^−1^ of either HPEG-NPs or HPEG-PH20-NPs with enzyme activity of 10 U mL^−1^ for 0, 1, 2, and 4 h. HA, nuclei, and DiD-labeled NPs were shown in green, blue, and red, respectively. The signal included both internalized particles and particles bound on cell surfaces (Scale bar: 50 μm). **d** In vivo tumor growth inhibition curves for 4T1 tumor-bearing mice that were treated with either saline, free DOX, DOX-HPEG-NPs or DOX-HPEG-PH20-NPs. The dose of DOX was 2 mg kg^−1^ for free DOX and DOX-encapsulated NPs. Values indicate mean ± SD (n = 6). **P < 0.01. Black arrows indicate the time of injection. **e** Survival rate plots show the percentage of animals remained alive in the study. Mice were sacrificed and were no longer counted for survival rate when their tumor size exceeded 2000 mm^3^. **P < 0.01. **f** TUNEL staining of sectioned tumor tissues that were collected after the completion of all doses for the groups treated with saline, free DOX, DOX-HPEG-NPs, and DOX-HPEG-PH20-NPs. Green, TUNEL; blue, nuclei. Scale bar: 100 μm. **g** Staining of sectioned tumor trusses that were collected 24 h post the administration of saline or NPs. The mice were *iv* injected with saline or DiD-labeled NPs on day 9 post 4T1 cell inoculation. Left column showed the staining of CD31 (green, representing blood vessels) and the distribution of NPs (red). The middle column showed the HA staining and the right column showed the α-SMA staining for the four study groups. Scale bar: 50 μm (left); 200 μm (middle); 100 μm (right). Reproduced with permission from Ref. [187]

Since the major matrix components include fiber, collagen, and hyaluronic acid (HA) [180, 181], direct degradation of these matrix components is another strategy to disrupt the physical barrier, among which the hyaluronidase-mediated HA degradation has received maximum attention [182–184]. Several studies have reported that the direct injection of hyaluronidase into tumors can effectively degrade intratumor HA and increase tumor accumulation and penetration of various nanoparticles and the levels of antigen-specific T cells at tumor sites [185, 186]. Furthermore, to prevent unnecessary degradation of a large amount of HA, thus potentially improving tumor progression, Zhou et al*.* conjugated hyaluronidase to the surface of PLGA nanoparticles through the click reaction to only degrade the matrix during nanoparticle diffusion [187] (Fig. 8). The study reported that covalently conjugated rHuPH20 was more efficient than free rHuPH20 in enhancing the nanoparticle diffusion in the matrix, thus inhibiting the growth of aggressive 4T1 tumors at a low drug dose. Moreover, another study used an exosome surface-displaying method to generate a naturally derived GPI‐anchored PH20-harbored exosome to address the limitations of enzyme immobilization-induced activity decline [188]. Similarly, exosome surface‐linked hyaluronidase displayed higher activity in cancer therapy than the previously assessed recombinant PH20 proteins. Furthermore, Zinger et al*.* reported a 100-nm liposome encapsulating collagenase, called collagozome [189]. Once being pretreated with the collagozome, the level of fibrotic tissue in the pancreas was reduced from 12.8 ± 2.3% to 5.6 ± 0.8%, thus increasing the drug penetration into the pancreas and initiating pancreatic ductal adenocarcinoma (PDAC) treatment.

#### Nanoparticle Penetration

**Fig. 9:**
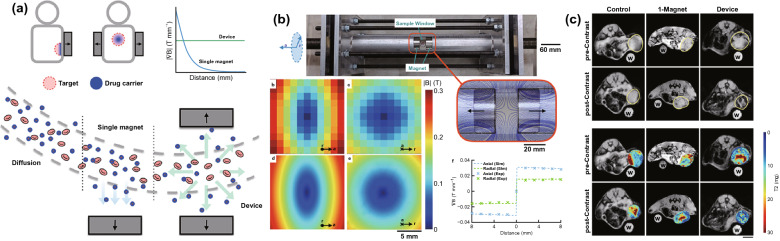
**a** Magnetic device, comprising two oppositely polarized magnets, enhances magnetic drug targeting in deep tissues. **b** Magnetic device contains a sharp zero point surrounded by constant field gradients. **c** Pre- and postcontrast images have similar signal distributions in the control. However, mice that have been exposed to the device show much less T2 signal (hypointensity) postcontrast. Water control labeled “w”. Scale bar = 5 mm. Reproduced with permission from Ref. [211]

Irrespective of the normalization strategy or matrix degradation approach, during the initial dosage, the already existing high interstitial fluid pressure [190], dense extracellular matrix [191], and tightly packed tumor cells [192] substantially limit the nanocarrier infiltration from the perivascular regions to distal cells [193, 194]. Thus, along with the adequate tumor accumulation, deeper tumor delivery of the formulation is required [195–198]. Most recent studies on penetrating nanoparticles are focused on reductions in particle size or charge reversal in response to TME-related factors (such as lower pH [199], hypoxia [200] and higher ROS level [201], rich GSH [202], and various enzymes [203–205]) or exogenous physical interventions (including laser irradiation [206], ultrasound [207], and thermal treatment [208]). Considering strategies to decrease the particle size, first is a complete nanocarrier with a smaller size, such as albumin [209], gold nanoparticles [202], nano-dots [208], and PAMAM [199], on which the cargo loading is easy. Thereafter, cross-linking of this nanocarrier together via various stimuli-responsive cleaved chemical bonds to generate a larger nanocarrier-aggregate with a suitable particle size of approximately 100–200 nm for long-term blood circulation, which could be dissociated into smaller drug-loaded segments on being triggered by certain stimuli at tumor sites, thereby achieving tumor penetration. Second, in some studies, ultra-small nanoparticles (about 5 nm) into a porous nanocarrier with a larger size of approximately 100–200 nm and then usually a biofilm was used to prevent the cargo leakage during the delivery [210]. Until triggered on by pH or near-infrared irradiation, the nano-cargo was released and infiltrated deep into the tumor. Charge reversal is similar to a process of protection and de-protection, wherein PEG [203, 206] or zwitterion [31, 205] is the commonly used disguise for shielding the inner positive charge of nanoparticles along with the introduction of stimuli-responsive cleaved chemical bonds. Upon pH, enzyme, or irradiation activation, the shielding part was eliminated, to result the cationic conjugate assisted the tumor infiltration of the remaining cargo-encapsulated nanoparticles. In particular, Zhou et al*.* reported that cationization in situ could effectively facilitate nanoparticle penetration across multiple cell layers through caveolae-mediated endocytosis and transcytosis pathways [205]. Along with the aforementioned two major categories, Liu et al*.* developed a device comprising two oppositely polarized external magnets facilitating magnetism-guided penetration of magnetic nanoparticles into deeper tumors, displaying a fivefold increase in the penetration rate than enhanced permeability and retention effect (EPR) [211] (Fig. 9). Furthermore, Lee et al*.* used host tumor-recruiting CD11b^+^ myeloid cells as a second active vector [212], and through an immune recognition reaction and bio-orthogonal click chemistry in vivo, tetrazine-functionalized drug-loaded nanoparticles might be associated with the second vector, then following myeloid cells through the depth of tumors.

#### Hypoxia Alleviation

**Fig. 10:**
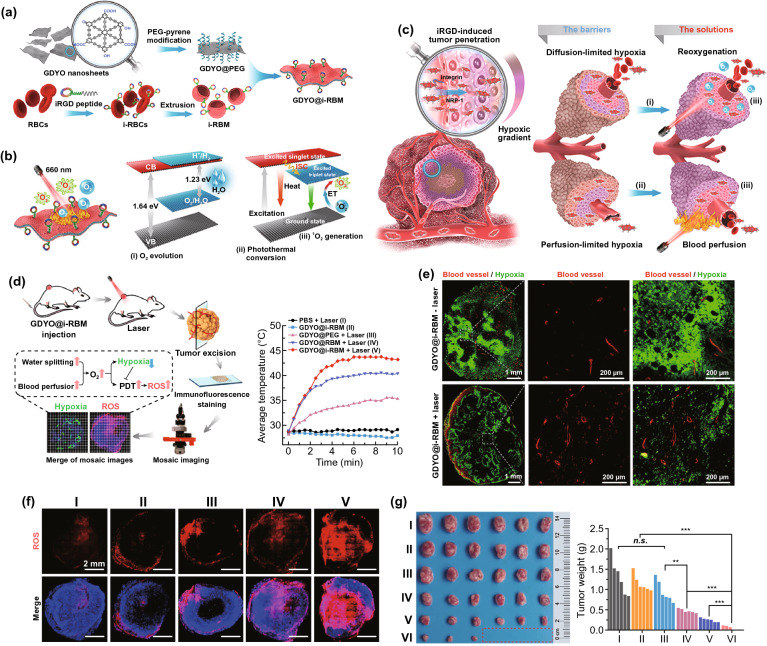
**a** Schematic illustration of the synthetic process of GDYO@iRBM. GDYO@i-RBM was obtained by coating PEG functionalized GDYO nanosheet with i-RBM, which were extracted from iRGD anchor-modified RBCs followed by extrusion through a porous membrane. **b** Schematic illustration of the working principles of GDYO@i-RBM.** c** i-RBM on the surface of GDYO facilitates the accumulation and deep penetration in the tumor. Meanwhile, under 660 nm laser irradiation, O_2_ evolution and hyperthermia caused by GDYO can overcome O_2_-diffusion-limited and perfusion-limited hypoxia barriers and lead to efficient PDT ablation of tumors. **d** Schematic illustration showing the evaluation of hypoxia and ^1^O_2_ levels in tumor, and temperature increase of the tumors upon irradiation. **e** Hypoxia immunofluorescence and vessel morphometric analyses of tumor slices. The blood vessels and hypoxia regions were stained with anti-CD31 antibody (red) and hypoxia probe (green), respectively. **f** Representative ROS fluorescence images of tumor slices. **g** Photos and weight of the tumor tissues obtained on day 22 post-treatment. Reproduced with permission from Ref. [224]

Hypoxia is distinctly associated with stromal improvement [213]. For oxygen generation, endogenous H_2_O_2_, as a tumor metabolite present at high levels (ranging from 100 μM to 1 mM) owing to excess ROS generated under hypoxia [214–216], can be used as an important source of O_2_. Apart from catalase [217], various inorganic H_2_O_2_ catalysts have been developed as nanocarriers for in situ catalytic H_2_O_2_ decomposition to O_2_ and hypoxia alleviation, e.g., MnO_2_ [218], copper/manganese silicate [219], Mn-carbon dots [220], porous platinum nanoparticles [221], Pt nanozymes [222], and Prussian Blue nanoparticles [223]. Moreover, Jiang et al*.* reported that H_2_O is another potential source of O_2_ when catalyzed by biomimetic ultrathin graphdiyne oxide (GDYO) nanosheets through near-infrared irradiation [224] (Fig. 10). For the oxygen delivery strategies, perfluorocarbons (PFCs) [225–228] and hemoglobin (Hb) [229–232] were two frequent categories of high oxygen-affiliative materials used as nanocarriers for oxygen loading, which were functionalized and encapsulated into liposomes or biomimetic membrane-camouflaged nanoparticles to facilitate oxygen delivery to hypoxic tumor sites with a low partial pressure of O_2_ and alleviating hypoxia to reinforce the efficacy of various anticancer modalities. Recently, through catalase-triggered H_2_O_2_ decomposition-dependent O_2_ generation, Song et al*.* developed a two-dose schedule to sequentially deliver catalase and exogenous H_2_O_2_ into hypoxic areas through well-established liposomes, which could decompose H_2_O_2_ into oxygen in tumors and were suitable for clinical translation for cancer radio-immunotherapy [233]. Furthermore, nanoparticles comprising CuO@ZrO_2_ [234], Au_2_O_3_ [235], or CaO_2_ [236, 237] were also used as oxygen carriers recently, which could be activated through microwave treatment or a reduction in pH, thereby producing O_2_ for the hypoxia alleviation.

### Strategies for Rescuing T cell Exhaustion

#### Characteristics of T Cell Exhaustion in Cold Tumors

Immunosuppressive cells (primarily MDSCs [238], TAMs [239], and Tregs [240]), which are largely recruited to solid tumors under the influence of hypoxic stress [241–243], play an equally crucial role in generating an immunosuppressive microenvironment as factors affecting T cells homing to cold tumors [244]. MDSCs are a heterogeneous population of myeloid cells including immature myeloid cells and myeloid progenitor cells and accumulate in tumor regions under the influence of various tumor-secreting factors including cyclooxygenase 2 (COX-2), granulocyte–macrophage colony-stimulating factor (GM-CSF), VEGF, TGF-β, and IL-6 [238]. Similarly, because of the signaling pathways associated with these factors, most of which converge on the Janus kinase (JAK) protein family members and signal transducer and activator of transcription 3 (STAT3), MDSCs are expanded and activated to exert remarkable immunosuppressive effects in the TME [238, 245, 246]. These functions include (i) the activation of arginase (ARG)-1, deprivation of *L*-arginine, which are not produced by T cells and critical for T cell proliferation and anti-tumor responses [238, 247]; (ii) the activation of nicotinamide adenine dinucleotide phosphate oxidase (NOX) and inducible NO synthase (iNOS), producing ROS and NO, respectively, leading to the oxidation of chemokines essential for T cell migration and nitration of T cell receptor (TCR)-induced reduction of antigen recognition and subsequent apoptosis in T cells and NK cells [248–250]; (iii) reinforcing TGF-β and IL-10 production and PD-L1 up-regulation, disrupting DC differentiation, migration, and antigen presentation, and inducing TAF activation and ECM deposition in conjunction with matrix metalloproteinase production [245, 251, 252]; (iv) up-regulation of angiogenic factors including VEGF, basic fibroblast growth factor (bFGF), and platelet-derived endothelial cell growth factor (PD-ECGF), thus promoting tumor neovascularization [253, 254]; (v) activation of regulatory T cell (Treg) differentiation through cytokine production or through direct cell–cell interactions [238, 241].

Once immune cells were recruited to the tumor lesions, macrophages are extraordinarily abundant and are present in all stages of cancer progression under a gradient of tumor-derived chemo-attractants including CCL-2, tumor necrosis factor (TNF), IL-8, IL-6, VEGF-A, and CSF-1 [255, 256]. The HIF-1 pathway [257], PI3K-PTEN-AKT pathway [258, 259], and loss of serine/threonine liver kinase B1 (LKB1) [260] are the mechanisms involved in the recruitment and activation of tumor-associated macrophages (TAMs) into solid tumors, usually through CXCL12 and CCL2 engagement with their receptors CXCR4 and CCR2, respectively. Other than normal macrophages, the potential of TAMs to present tumor-associated antigens is decreased, instead of several pro-tumoral M2 phenotype-associated functions, including angiogenesis, matrix remodeling, immunological suppression, and tumor metastasis [261]. For example, TAMs participate in pro-angiogenic phenomena by expressing angiogenic factors including Wnt7b, TIE2, and thymidine phosphorylase (TP), thus stimulating vascular endothelial cells to produce VEGF, leading to an angiogenic switch [255]. Expression of PD-1 and CTLA-4 ligands can suppress the cytotoxic functions of T cells and NK cells [261, 262]. Furthermore, TAMs can up-regulate the ligands for death receptors including TRAIL and Fas to induce apoptosis in targeting cells [261, 263]. Secreted cytokines TGF-β and IL-10 inhibit the effector functions of T cells [264, 265] and induce the release of chemokines including CCL5, CCL20, and CCL22 for Treg recruitment [266, 267]. Beyond that, TAMs can produce matrix metalloproteases (e.g., MMP2 and MMP9) and factors (e.g., TGF-β, PDGF, IL-6, urokinase plasminogen activator (u-PA), and tissue-type plasminogen activator (t-PA)) to degrade the ECM for tumor invasion and migration [268, 269].

Apart from the tumor-infiltrating CD8^+^ T cells (major in hot tumor), tumor cells, M2-like TAMs, and MDSCs can facilitate Treg tumor infiltration by increasing CCL22 secretion in cold tumors [270]. Furthermore, Tregs can actively produce IL-10 and TGF-β, thus suppressing cytotoxic T cells and immune tolerance [271]. In addition, Tregs can exhibit immunosuppressive functions through direct mechanisms including IL-2 deprivation, CD39/CD73-mediated adenosine generation, and competition with CD28 (a co-stimulator on CTLs) for binding to CD80/CD86 on APCs by inducing CTLA-4 (a co-inhibitor) expression [3].

#### MDSC-Targeting Treatments

Among the immunosuppressive cells in cold tumor lesions, MDSCs have been considered as the most versatile cells [272, 273], for which plenty of approaches have been developed to abrogate its suppressive activity in vivo, including the following: (i) MDSC elimination, (ii) blockade of MDSC recruitment, (iii) inhibition of MDSC suppression, and (iv) facilitating MDSC differentiation. Unfortunately, nanotechnology has not been extensively applied in this field, and most treatment strategies have focused on the development of chemicals or antibodies [273–275]. Therefore, a brief description of therapeutic agents has been provided below, facilitating the guidance of the development of MDSC-targeting nanoformulations.

Low-dose chemotherapy, including gemcitabine [276] and 5-fluorouracil [277], has proven effective in depleting MDSC populations in tumor bearers, and tyrosine kinase inhibitors (including Sunitinib [278]) have successfully eliminated MDSCs in cancer patients by blocking VEGF, STAT3, and c-KIT signaling. Furthermore, regarding the blockade of MDSC recruitment, antagonists of chemokines (CCL2, CCL5, CSF-1, and G-CSF) and their receptors (CXCL2, CCR5, and CSF-1R) engaged in tumor chemotaxis of MDSCs have been identified as strategically promising therapeutic agents to inhibit MDSC migration to tumor lesions to restrict tumorigenesis [279–282], and most of these agents have been reported in previous clinical trials [283], *e.g.,* phase II clinical trials on CXCR2 antagonist Reparixin for TNBC (NCT02370238) and phase 1 clinical trials on CCR5 antagonist Maraviroc for metastatic colorectal cancer (NCT01736813). Moreover, STAT3 inhibitor (AZD9150) [284], reactive nitrogen species (RNS) inhibitor (AT38) [285], nitroaspirin (NCX4060 and NCX4016) [286], phosphodiesterase-5 inhibitors (sildenafil) [287, 288], triterpenoids (CDDO-Me) [246], COX-2 and PGE2 inhibitors (Celecoxib, ASA) [289, 290], HDAC inhibitor (Entinostat) [291], and very-small-sized proteoliposomes (VVSP) [292] have effectively attenuated the potent immunosuppressive functions of MDSCs to reconstitute T cell responses and the success of immunotherapy. Finally, differentiation of suppressive MDSCs into mature myeloid cells (including macrophages and DCs) through treatment with Vitamin D3 [293], all-trans-retinoic acid (ATRA) [294], taxanes (docetaxel and paclitaxel) [295], TLR9 activation (CpG) [296], curcumin [297], whole-glucan particles (WGP) [298], and casein kinase inhibitor (tetrabromocinnamic acid) [299] can overtly modulates MDSCs and decrease the tumor growth in tumor-bearing mice and cancer patients.

**Fig. 11:**
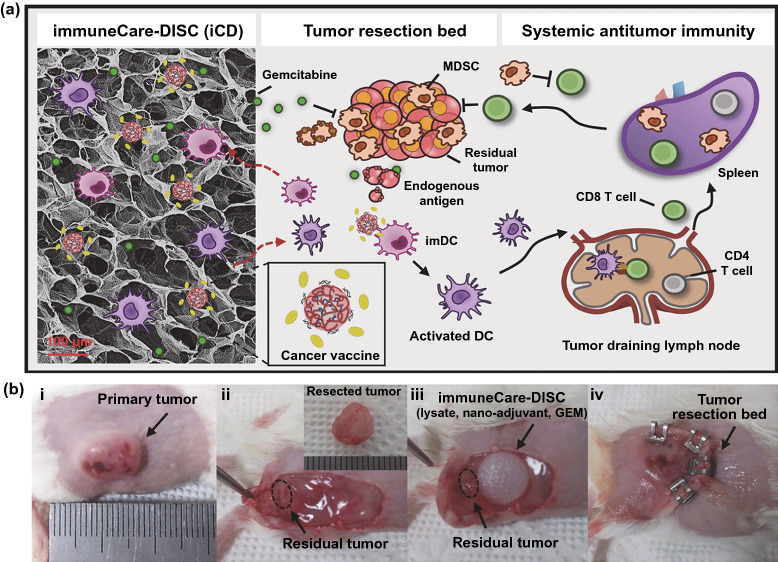
**a** Schematic diagram showing the design of the iCD, which codelivers GEM, as an MDSC-depleting drug to revert the immunosuppressive microenvironment, and cancer vaccines consisting of whole tumor lysates and nanoadjuvants carrying TLR3 agonists, to provide immunostimulation and elicit an anti-tumor immune response. Release of the vaccine induced the infiltration, activation, and homing of DCs to the lymph nodes, which initiated an antigen-specific adaptive immune response in a host environment where tumor-induced immunosuppression was depleted by MDSCs. **b** Implantation approach: (i) surgery was performed after the tumor volume reached ≈300 mm3, (ii) tumor dissection mimicking incomplete tumor removal (≈90% of primary tumor was excised), (iii) implantation of the iCD containing GEM and cancer vaccines, (iv) wound closure. Reproduced with permission from Ref. [300]

As expected, several nanotechnology-based strategies have been developed for MDSC-targeting therapies, such as gemcitabine-loaded nanocarriers to eliminate MDSCs [205, 300–303] (Fig. 11), hypoxia alleviation-mediated MDSC elimination by platelet membrane-based co-encapsulation of metformin and IR780 [304], phosphoinositide-3-kinase-γ (PI3K-γ) inhibition-mediated MDSC remodeling by IPI-549-loaded targeted polymeric nanoparticles [305], disruption of MDSC expansion by pseudoneutriphil cytokine sponges [306], co-delivery of RNAi and chemokines in polyarginine nanocapsules for MDSC modulation [307], and inhibition of MDSC recruitment by micellar hypotoxic low molecular weight heparin-tocopherol succinate nanoparticles [308].

#### TAM-Targeting Treatments

**Fig. 12:**
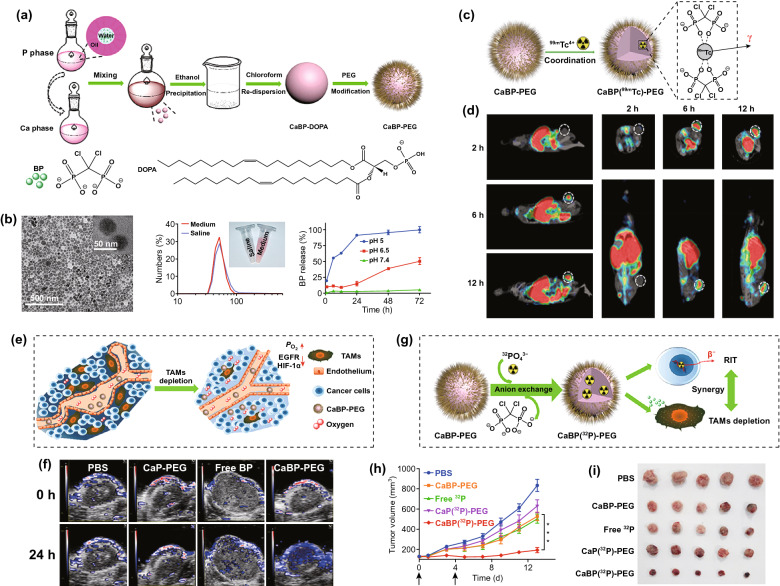
**a** Schematic illustration to show the preparation of CaBP nanoparticles. **b** TEM image and dynamic light scattering data and photo of CaBP-PEG nanoparticles in PBS and cell culture medium buffer, as well as release of BP from CaBP-PEG nanoparticles under different pH values. **c** Scheme illustrating the ^99^mTc radiolabeling of CaBP-PEG nanoparticles for tracing the in vivo fate of nanoparticles. **d** SPECT images of mice at 2, 6, and 12 h after intravenous injection of CaBP(^99^mTc)-PEG nanoparticles. Tumors of mice are highlighted by the dotted circles. **e** Scheme showing the normalized tumor vasculature, enhanced perfusion, and relieved tumor hypoxia after TAM depletion.** f** Scheme illustrating CaBP(32P)-PEG nanoparticles for synergistic combination RIT with TAM depletion. **g** Photoacoustic imaging showing tumor saturated O_2_ levels of mice after i.v. injection of PBS, CaP-PEG, free BP, or CaBP-PEG nanoparticles. **h** Tumor volume curves of mice with various treatments. Doses for each injection: 100 μCi of 32P, 200 μg of BP. The mice were treated twice at day 0 and 4 (black arrows). **i** Photographs of tumors collected on the 14th day after the first treatment. Reproduced with permission from Ref. [320]

Three main categories of TAM-targeting therapies have been reported so far, including chemotaxis blockade, M2-like TAM elimination, and M2-TAM repolarization, of which the delivery via nanocarriers has been extensively studied [309–312]. Peripheral monocytes, TAM predecessors, are recruited into the tumor lesions through the engagement of CSF1/CSF1R [313] and CCL2/CCR2 [310]; hence, the blockade of these factors might potentially reduce the accumulation of TAMs in tumors, thereby overcoming the TAM-related immunosuppression and enhancing tumor-specific T cell responses. For example, Shen et al*.* generated monocyte-targeting cationic nanoparticles to deliver CCR2 siRNA for inhibiting the recruitment of Ly6C^hi^ monocytes by blocking CCL2-CCR2 pathways [314, 315]. Furthermore, Ramesh et al*.* developed a CSF1R- and SHP2-inhibitor-loaded nanoparticle for reinforcing cytotoxic activity and phagocytosis of TAMs [316]. Herein, the CSF1R-inhibitor was used for inhibiting the recruitment and differentiation of TAMs into the M2-like phenotype, and the SHP2-inhibitor could potentially increase TAM phagocytosis by suppressing the downstream ‘eat-me-not’ signals of the CD47-SIRPα axis [61]. Regarding M2-TAM elimination, induction of TAM apoptosis via nanocarriers is a common approach [309]. Bisphosphonates (BPs) constitute first-line low-cost agents for treating metabolic bone diseases and exhibit selective toxicity to TAMs [317–319]. Tian et al*.* mineralized BPs with Ca^2+^ to synthesize a BP-loaded nanoparticle and then functionalized the nanoparticle with poly(ethylene glycol) [320]. Furthermore, to endow the BP nanoparticle with theranostics, a single-photon-emission computed tomography (SPECT) contrast element, ^99m^Tc, was introduced into the system via coordination bonds. As revealed through the SPECT imaging, BP nanoparticles exhibited efficient tumor retention post-injection (*i.v.*) and facilitated effective depletion of TAMs within the tumor (Fig. 12). Moreover, another attractive strategy for tumor eradication is reversing TAM polarization from an immunosuppressive M2 to a tumoricidal M1 phenotype [321]. Effective approaches in animal models have involved TLR7/8 ligation with R848-loaded β-CD nanoparticles (CDNP-R848) [322], up-regulation of pro-inflammatory signals (iNOS and TNF) with liposome-encapsulated zoledronate acid treatment [323], and nanoparticle-mediated delivery of M1-like TAM-related active microRNAs, such as miR-125, for TAM reprogramming [324, 325]. Recently, several independent studies have consistently reported that superparamagnetic iron oxide nanoparticles (SPIONs) can also reorient M2-like TAMs into an M1-like phenotype through multiple approaches [326–329], e.g., interferon regulatory factor 5 (IRF-5) signaling-mediated M1 polarization and M2-like phenotype-related arginase-1 down-regulation [326]. Moreover, surface-mannosylation has been widely used to harness TAM-targeting nanocarrier systems [218, 330–333].

#### Treg-Targeting Treatments

Tregs represent the third major cell type contributing to the immunosuppressive TME in cold tumors [334, 335]. In fact, the interactions among the three categories of inhibitory cells are closely associated [241, 243, 336]. Therefore, regardless of immunotherapy aimed at MDSC or TAM modulation, the quality and number of Tregs would also be simultaneously affected [241, 337–339]. Furthermore, antibodies against surface molecules (including CD25 [340], CCR4 [341], CTLA-4 [342], OX40 [343], and GITR [344, 345]) exhaust Tregs in various tumor models, and molecules (such as cyclophosphamide [346] and Raf-kinase inhibitor sorafenib [347]) can also preferentially deplete Tregs upon systemic administration. As expected, the application of nanotechnology in Treg-targeting treatments was in accordance with the two aforementioned categories. Li et al*.* developed CTLA-4-siRNA loaded PLGA nanoparticles to successfully deliver CTLA-4-siRNA into both CD4 + and CD8 + T cell subsets at the tumor sites, thereby down-regulating CTLA-4 in activated T cells and enhancing anti-tumor immune responses [348]. Similarly, Liu et al*.* encapsulated anti-CTLA-4 into PLGA nanoparticles for protein-protective delivery [349]. Furthermore, strategies involving the loading of cyclophosphamide into vehicles including in situ-developed fibrin scaffolds [350] or engineering of PD-1-presenting platelets [351] exhibited selective depletion of Tregs and effective postsurgical prevention of cancer relapse in combination with anti-PD therapy.

### Strategies for Treatment of Pseudo-Cold Tumor

Although the positive PD-L1 expression and T cell infiltration have been considered as a predictor of effective outcome of PD-1/PD-L1 antibody therapy, there are a growing number of patients with PD-L1 overexpression that cannot benefit from the anti-PDs treatment [352]. Among these patients, some patients showed primary resistance to treatment like having a cold tumor with low mutational burden, infiltrated immunosuppressive cells or up-regulated immunosuppressive factors, and others usually developed an adaptive resistance to continuation of PD-1/PD-L1 blockade therapy after a period of robust initial response. Since they are also ineffective against anti-PDs therapy, but different from the cold tumors in terms of TIME, we refer to them as pseudo-cold tumor.

In regard to primary resistance, several studies proposed that genetic mutations might be responsive for the primary resistance of PD-L1-positive patients [353]. In a clinical study on patients with non-small cell lung cancer (NSCLC), researchers found that patients with EGFR mutations and ALK rearrangements showed a low CD8^+^ T cell infiltration-associated poor response to anti-PDs therapy despite a high rate of PD-L1 expression, which implied that the expression of PD-L1 could also be constitutive rather than just responding to inflammatory stimuli [354]. However, it is unfortunate that the mechanism of action between theses mutations and apparent T cell exclusion remains unclear. As far as we know, it would be a promising approach for the reversal of primary resistance to combine the therapeutic strategies for cold tumors and mutant protein-targeted therapy. Besides, it has been identified that there were also various immunosuppressive cells such as MDSC, TAMs, Tregs existing to impair the functionality of anti-tumor T cells. Hence, the previously mentioned strategies for rescuing T cell exhaustion are applied.

Recently, in addition to PD-1, several biomarkers have been identified as alternative checkpoint receptors that are up-regulated and involve in T cell dysfunction during anti-PDs therapy, resulting in adaptive resistance [355]. These include cytotoxic T-lymphocyte antigen 4 (CTLA-4), T cell immunoglobulin and mucin domain-containing-3 (TIM-3), T cell immune receptor with Ig and ITIM domains (TIGIT), lymphocyte activation gene-3 (LAG-3), V-domain Ig suppressor of T cell activation (VISTA), and so forth. For these patients, if possible, a needle biopsy and in-depth immune analysis of the new or increasing site of resistance can first be performed to determine the mechanism of resistance and treat it according to the cause. Nowadays, it is a hot-spot to develop effective antibodies against these targets, as there are numerous cases of clinical trials underway for each one [356].

Furthermore, some researchers have proposed that the outcome of PD-L1 antibody therapy greatly relies on the distribution of PD-L1 [357]. According to PD-L1 distribution, the PD-L1 can be classified into four formats: serum PD-L1 (sPD-L1), membrane PD-L1 (mPD-L1), cytoplasm PD-L1 (cPD-L1), and nuclear PD-L1 (nPD-L1). Among them, mPD-L1 is the major format that can bind to its receptor (PD-1) to enable immunologic tolerance, which can be blocked by utilization of PD-L1 antibody. However, as for cPD-L1 and nPD-L1, due to their intracellular location, the blockade of cell membrane can theoretically impair the efficacy of PD-L1 antibody against these formats. Considering the translocation of PD-L1s, the cPD-L1 and nPD-L1 may be the reservoir for mPD-L1 to be translocated onto the surface when treated anti-PD-L1s have been eliminated, resuming the immune escape. At the same time, the mPD-L1 can be also translocated into the cytoplasm to be cPD-L1 to avoid being tracked during the anti-PD-L1 treatment. In a clinical study on papillary thyroid carcinoma [358], patients with cPD-L1 up-regulation showed a shorter disease-free survival compared to those lack of cPD-L1, which highlighted the function of cPD-L1. Therefore, the positive expression of intracellular PD-L1 could be an underlying incentive for adaptive resistance and therapeutic strategies, such as PD-L1 gene silencing, sPD-1 mRNA or plasmid transfection, and chemical inhibitor treatment, that can suppress the functions and sources of entire PD-L1 may be advantageous over antibody blockade. In this regard, up to now, there have been various and countless nanocarriers developed to enable efficient gene delivery or controlled delivery of small molecule drugs alone or in combination, which is thus not described in this article.

## Conclusion and Perspectives

As described in this review, there are three major challenges in anti-cold tumor immunotherapy: T cell priming inhibition, T cell exclusion, and T cell exhaustion. Nanomedicines, within the capability of cargo protection and controlled release, as well as the designability for tumor or immune cell targeting, have shown effective therapeutic outcomes in reversing these limitations. In regard to normalize T cell priming, it is critical to recover the antigen presentation of APCs. Strategy that delivering STING agonists (represented by cGAMP) by DC-targeting nanocarrier can significantly increase the accumulation of cGAMP in DCs, reduce the off-target side effect, and accelerate the DC maturation. Meanwhile, stimuli-responsive nanomedicines can enable the loading adjuvants and antigens controllable release in cell as needed, so as to exert their capability of DC activation preferably. Secondly, the deposition of extracellular matrix in cold tumor lesions builds a physical barrier to exclude T cell infiltration. Codelivery of therapeutic agents with matrix catabolic enzymes or deposition inhibitors via nanomedicine has shown capability to loose extracellular matrix from the lateral side and facilitate agents and T cell penetration. In addition, nanomedicines with TME-responsive size-change or charge-shift can escort their cargos across the matrix barrier and arrive at the deeper tumor, resulting in appropriate microenvironment for T cell infiltration. Furthermore, the designability of nanomaterials endows the nanomedicines with the capability of targeting multiple immunosuppressive cells. Although the application of nanomedicines has accelerated the research on anti-cold-tumor immunotherapy and holds enormous promise, we are still in initial stage of the clinical translation of nanomedicines for immunotherapy, and there are several overlooked key problems in preclinical research which have to be addressed. First, do we employ the right tumor-bearing animal models? It is a fundamental but critical question for therapeutic evaluation of nanomedicines developed for anti-cold-tumor immunotherapy in preclinical studies. At present, more and more scholars have highlighted that the right therapeutic regimens should be applied in the right patients/tumors when possessing the clinical trials [40]. However, many current preclinical studies of cold tumor treatment were carried out on inappropriate animal models, for example, a common hot tumor-bearing model (subcutaneous melanoma) was widely used as a cold tumor model to investigate immunomodulatory effects of ICD therapy, as well as even though a right cold tumor cells chosen, usually a subcutaneous tumor-bearing model was employed for research rather than the in situ tumor-bearing model, which is also not advisable. In particular, as for pancreatic cancer, a well-known cold tumor, where the fibrosis of pancreatic stellate cells in pancreas is also one of the critical factors involved in cold TIME, is of little significance to investigate the effect of anti-tumor immunotherapy on subcutaneous tumor-bearing model, instead, resulting in a waste of resources.

However, limited by the heterogeneity between individual animals and current clinically disjointed methods of diagnosis and evaluation in preclinical studies, there exists difficulty in defining a right animal model. At least, a real-time biopsy method, which can monitor the change of TIME before and after treatment in the same animal by flow cytometry or immunofluorescence staining, should be introduced into preclinical trials to replace current one-time terminal evaluation. In addition, a tempo-spatio testing standard should be established, such as biopsy of experimental animals, the appropriate time spot to test the T cell levels, and quantity of the T cells detected would be considered as a primal cold tumor or a fired-up cold tumor.

Second, it’s a cliché topic, drug safety. In addition to efficacy, the safety of new drug is another key determinant of whether the new drug is eligible for an investigation new drug (IND) approval from FDA, as well as the goal of phase I clinical trial is to determine the safety profile and pharmacology of new drug [359]. Although the damage to metabolic organs, such as liver and kidney, has been routinely evaluated by hematoxylin–eosin (H&E) staining or by further blood examination of aspartate aminotransferase (AST), alanine aminotransferase (ALT), etc., there are few other toxicity assessment of chemicals or nanomedicines (such as ototoxicity [360]), as well as the investigation of immune-toxicity (such as the detection of IL-17 [51]), that have been implemented on animal model. Therefore, it is essential and necessary to establish a sound evaluation mechanism for adverse immune reactions in preclinical study about the immunotherapeutic agents. Besides, the major feature of clinically validated nanomedicines for immunotherapy is to reduce the immunogenicity and improve the stability of immunomodulators, for example, the PEGylated IFN alpha-2a protein for Hepatitis C (Pegasys®, Genentech). With regard to nanomedicines composed of nanomaterials possessing the capability to activate the immune systems, we need to pay more attention to their potentially increased risk of adverse immune reaction during treatment, perhaps the applications of these nanomaterials could be suspended for a while.

Third, as for those ‘easy’ drugs that already have been approved and marketed, eutherapeutic and low-toxicity nanoformulations, such as paclitaxel (Abraxane) and doxorubicin (Doxil), would be extremely hard to develop new formulation to heighten the clinical benefits; instead, there have been many clinical trials focused on expanding the spectrum of approved nanomedicines for anti-tumor immunotherapies. Hence, a potential is to use the same approved nanomaterials, such as liposome and albumin, to prepare similar nanoformulations of new drugs, or to adjust the minimal structural alterations on approved nanomaterials to achieve our goal, such as pH or enzyme-responsive drug release, and targeted modification. Meanwhile, the studies on several promising nanoformulations, including polymeric micelle, iron oxide nanoparticle, gold nanoparticle, and PLGA nanoparticle, should be continued to obtain enough data to support the safety and efficacy evaluation.

In summary, the application of nanomedicines has shown favorable results in enhancing or even dramatically improving the efficacy of immunotherapeutic agents, as well as reducing toxicity. Currently, although there is not likely to be a mass clinical translation of nanomedicines owing to the immature technology for monitoring the fate of nanomedicines in vivo, thousands of different types of nanomedicines have been accumulated in this field waiting for a qualitative leap, once a sound evaluation system is established (Tables 1, 2, 3, 4, 5, 6).

**Table 1:** Characteristics of T cell priming inhibition in cold tumors

Categories	Components	Mechanism	Refs.
Inhibition of T cell priming	Disruption of APC recruitment	Wnt-β-catenin pathway-mediated CCL4 down-regulation	[22]
		Cox1/2-PGE2 pathway-mediated CCL5 and XCR1 down-regulation	[56]
	Disruptions of APC function	Cytokines (M-CSF, TGF-β, IL-6, IL-10)	[57–60]
		MYC-associated CD47 up-regulation	[61]
	Decreased immunogenicity	Impairment of antigen processing through alterations in proteasomal or post-proteasomal machinery	[63, 64]
		Regulation of antigen presentation through MHC-I mutations	[65, 66]
		Antigenic discontinuum driven by KRAS or BCR-ABL1	[67–70]

**Table 2:** Advanced application of nanomedicines for T cell priming resumption

	Target	Mechanism	Vehicle/agents	Combination	Models	Refs.
DC recruitment and its functional enhancement	CCL4	Stromal delivery of CCL4	Fusion protein of CCL4 and the collagen-binding domain (CBD) of von Willebrand factor	Immune checkpoint blockade (ICB)	EMT6 and MMTV-PyMT breast cancer, CT26 and MC38 colon carcinoma, B16F10 melanoma,	[26]
	DC autophagy	In situ DCs manipulation	Nano-activator comprising the antigen peptide and autophagy-inducing peptide (Bec1)	\	B16F10-OVA tumor	[71]
	cGAS-cGAMP-STING	Enhancing cytosolic cGAMP release	Phosphatidylserine-coated liposome loaded with STING agonist cyclic guanosine monophosphate–adenosine monophosphate (NP-cGAMP)	Radiotherapy (RT)	B16-OVA lung metastases, 4T1-luci lung metastases	[73]
		Promoting endosomal escape of cargo	pH-responsive cross-linkable polymersome	ICB	B16F10 tumor	[76]
		Rapid intracellular payload release	pH-sensitive acetalated dextran (Ace-DEX) polymeric microparticles (MPs)	Hemagglutinin	Lethal influenza	[77]
		Controlled release	Anti-parallel β-sheet nanofibrous hydrogels	\	Head and neck cancer	[78]
	Artificial DCs	Inheriting the function of DCs	DC membrane coating nanovaccine	\	Ovarian cancer	[84]
		Retaining the function of DCs	DC-derived microvesicles	Chemotherapy	B16F10 tumor	[85]
Synchronous delivery of adjuvants and neoantigens	CpG	Co-delivering with DNA-antigen peptide	Self-assemble lipid-DNA-peptide nano-aggregation (INA)	*p*OVA, *p*AH1, *p*TRP2	Melanoma (B16-OVA, B16-TRP2), carcinoma (CT-26-AH1)	[95]
		Delivering adjuvants to endosomes, antigen to cytosol	pH-sensitive polymer (MGlu-HPG)-modified liposomes	Ovalbumin (OVA), CTL epitope peptide	EG7-OVA tumor	[94]
	TLR7/8	Co-delivering peptide antigens and adjuvants	Self-assembling vaccine platform (SNP-7/8a)	aPD-L1	B16-OVA tumor, B16F10 tumor, TC-1 tumor, MC38 tumor	[96]
	mRNA-encoding antigen	Nucleotide-modified mRNA for mRNA protection	Lipid nanoparticle encapsulating nucleoside-modified mRNA and TLR4 agonist monophosphoryl lipid A (MPLA)	\	Healthy C57BL/6 mice	[91]
			mRNA Galsomes encapsulating nucleoside-modified mRNA and the iNKT ligand α-GC	aPD-L1	EG7-OVA tumor, B16-OVA tumor	[104]
	Lymph node targeting	Passive transport	Negatively charged 30 nm-sized lipid nanoparticles (LNPs)	\	Healthy female C57BL/6 J mice	[108]
		Albumin-based	Long-chain fatty acids coupled adjuvant	\	Healthy C57BL/6 mice	[112]
		Lymphatic vessel-expanded	Controlled NO release nanocarrier (SNO-NP)	\	Healthy female C57BL/6 J mice	[113]
ICD induction	Chemotherapy	Boosting out ROS	Tumor-targeting Pt‐prodrug-loaded Fe3O4 nanoparticles	Ferroptosis	4T1 tumor	[126]
	Photodynamic therapy	Combination of ICD with CD47 blockade	Tumor microenvironment‐activatable prodrug vesicles	CD47 blockade	CT26 tumor, 4T1 tumor	[52]
		Evoking superior ICD	AIE photosensitizers, TPE‐DPA‐TCyP	\	4T1 tumor	[131]
		Exhibiting 1.7-fold higher PDT efficacy than the PS	Photochromic metal–organic frameworks	\	\	[144]
		Combination of PDT and hypoxia-activated chemotherapy	Core–shell upconversion nanoparticle@porphyrinic MOFs (UCSs)	Tirapazamine, α-PD-L1	CT26 tumor	[145]
		Low-dose X-ray-induced PDT enhancement	Hafnium (Hf)-based nanoscale metal–organic framework (nMOF)	IDO inhibitors	U87MG tumor, PC-3 tumor, CT26 tumor	[147]
	Sonodynamictherapy	Co-delivery of R837	Clinically approved material comprising HMME/R837@Lip	Anti-PD-L1	4T1 tumor, CT26 tumor	[150]
		Co-delivery of O_2_	R780@O2-FHMON nanoparticle	\	PANC-1 tumor	[151]
		GSH exhaustion	Metabolism-engineered and SDT-based nanoplatform (Nb2C/TiO2/BSO-PVP)	\	4T1 tumor	[153]

**Table 3:** Characteristics of T cell exclusion in cold tumors

Categories	Components	Mechanism	Refs.
Blockade of T cell infiltration	Down-regulation of T cell homing factors	Histone modifications and DNA methylation down-regulating CXCL9 and CXCL10	[154, 155]
		IDH-1/2-mediated R-2-hydroxyglutatate generation inhibiting CXCL9 and CXCL10 production	[156]
		CXCL9 and CXCL10 down-regulation due to reduced type I IFN	[157]
	Physical barriers	Release of mitogenic and fibrogenic factors (VEGF, PDGF, TGF-β, and SHH) for TAF activation owing to KRAS or NF-κB mutations	[161–164]
		Positive feedback network of pro-inflammatory cytokines (VEGF and TGF-β) for ECM deposition	[165]
		Abundant ECM components (collagens, glycoproteins, elastin, and hyaluronan)	[165]
	Hypoxia	VEGF resulting from HIF-1α up-regulation inducing FasL/Fas-mediated T cell apoptosis	[166, 167]
		High VEGF-mediated increase in neovascular permeability inducing plasma protein extravasation for facilitating ECM deposition	[158, 168]
		VEGF expression-mediated defects in leukocyte adhesion molecules (ICAM-1/2, VCAM-1, and CD34)	[169]

**Table 4:** Advanced application of nanomedicines for overcoming T cell exclusion

Strategy	Target	Mechanism	Vehicle/agents	Combination	Models	Refs.
Stroma normalization	Vasculature normalization	Low dose of anti-VEGF treatment-related vasculature normalization	Anti-VEGF antibody (DC101)	α-CTLA-4, α-PD-1	Breast cancer (E0771, MCA-M3C, MCAP008), hepatocellular carcinoma (RIL-175)	[170]
	Chemokines	Inhibition of CXCR4/CXCL12 signaling	Plerixafor	ICB mixture of α-CTLA-4 and α-PD-1	Primary breast cancer, Liver and lung metastases breast cancers (E0771, MCa-M3C, 4T1)	[177]
		Suppression of TGF-β signaling	Tranilast	Doxorubicin, Abraxane, Doxil	MCF10CA1a human breast cancer, 4T1 tumor	[178]
	Pancreatic stellate cells (PSCs)	Regulation of PSCs	β-cyclodextrin (β-CD) modified liposome	Pirfenidone, gemcitabine	PSCs/Panc-1 pancreatic tumor	[179]
Matrix degradation	Hyaluronic acid (HA)	Tumor-local injection of HAase-related HA degradation	HAase/ ultrasensitive pH triggered charge/size dual-rebound P[(GP)D] nanoparticles (NPs), shPD-L1	\	B16F10 tumor	[185]
		Conjugated rHuPH20-mediated matrix degradation	rHuPH20-modified PEG-PLGA nanoparticles encapsulating doxorubicin	\	4T1 tumor	[187]
		Membrane‐anchored enzyme with high activity for HA degradation	GPI‐anchored PH20-harbored exosome encapsulating doxorubicin	\	PC3 tumor, 4T1 tumor	[188]
	Collagen	Collagen degradation	Collagenase-encapsulated liposome	Paclitaxel micelles	mCherry-labeled KPC orthotopic tumor	[189]
Penetrating-nanoparticle exploitation	Size reduction	pH-responsive size-switching	Platinum-prodrug conjugated polyamidoamine (PAMAM) dendrimers	\	BxPC-3 pancreatic tumor	[199]
		Hypoxia‐responsive dissociation	Human serum albumin (HSA)‐based nanosystem (HCHOA)	Oxaliplatin, photosensitizer chlorin e6	4T1 tumor	[200]
		Light-triggered ROS-responsive size-reducing	Hyperbranched polyphosphoester self-assembling nanoparticle	Chlorin e6, CPT	HT29 tumor	[201]
		Sequential pH and reduction‐responsive size reduction	Polymer and gold nanorod (AuNR) core–shell nanoplatform	Dox	MDA‐MB‐435 tumor	[202]
	DePEGylation	MMP‐2‐mediated PEG corona de-shielding	Temperature‐sensitive liposome (TSL) encapsulating photosensitizer pheophorbide, oxaplatin-prodrug, and DOX	Photodynamic therapy (PDT)	4T1 tumor	[203]
		NIR responsive dePEGylation	HTMP and iPHT coating up-conversion nanoparticles (UCNPs)	Dox	MCF-7 tumor	[206]
	Disassembly	Ultrasound (US) responsive disassembly	Janus Au‐MnO nanoparticles (JNPs)	Chemodynamic therapy (CDT)	MCF‐7 tumor	[207]
		Temperature‐sensitive disassembly	Self‐assembled temperature sensitive nanoclusters	Dox	4T1 tumor	[208]
	Transcytosis	Caveolae-mediated nanoparticle transcytosis	γ-glutamyl transpeptidase-responsive camptothecin–polymer conjugate self-assembling nanoparticles	CPT	HepG2 tumor, orthotopical BxPC-3-luci-GFP tumor	[205]
		Two oppositely polarized magnets-forced penetration	Superparamagnetic iron oxide nanoparticle (SPION) micelles	\	4T1 tumor	[211]
		In vivo bio-orthogonal click reaction-mediated conjugation to CD11b + myeloid cell	Tetrazines-functionalized drug-loaded nanoparticles	Dox	4T1 tumor	[212]
Hypoxia alleviation	Oxygen generation	Inorganic intracellular H_2_O_2_ catalysts	Man-HA-MnO2, copper/manganese silicate, Mn-carbon dots, porous platinum nanoparticles, Pt nanozymes, Prussian Blue nanoparticles	Dox, PDT, CDT, RT	4T1 tumor, MCF-7 tumor, H22 tumor, NCI-H460 tumor	[218–223]
		Catalase	CAT-encapsulated, Ce6-doped hollow silica nanoparticles (CAT@S/Ce6)	PDT	4T1 tumor	[217]
		Catalyzing water oxidation to release O_2_	Biomimetic ultrathin graphdiyne oxide (GDYO) nanosheets	PDT	EMT-6 tumor	[224]
		High oxygen-affiliative materials	Perfluorocarbons (PFCs) nanoparticle, hemoglobin (Hb)‐linked conjugated polymer nanoparticles (CPNs)	TPZ, Salmonella VNP20009,	CT26 tumor	[225, 229]
		Sequentially delivering catalase and exogenous H_2_O_2_	Well-established liposomes encapsulating H_2_O_2_ and catalase respectively	RT	4T1 tumor	[233]
		Inorganic oxygen generators	Core–shell CuO@ZrO_2_, Au_2_O_3_, CaO nanocomposites	Dox,	H22 tumor, U14 tumor,	[234–237]

**Table 5:** Characteristics of T cell exhaustion in cold tumors

Categories	Components	Mechanism	Refs.
T cell exhaustion	MDSCs	ARG-1 activation-mediated *L*-arginine deprivation	[238, 247]
		NOX and iNOS activation-mediated chemokine oxidation and TCR nitration	[248–250]
		Production of TGF-β and IL-10 and up-regulation of PD-L1	[245, 251, 252]
		Up-regulation of angiogenic factors (VEGF, bFGF, and PD-ECGF)	[253, 254]
		Activation of Tregs	[238, 241]
	TAMs	Expression of angiogenic factors (Wnt7b, TIE2, TP, and VEGF)	[255]
		Expression of PD-1 and CTLA-4 ligands	[261, 262]
		Up-regulation of TRAIL or Fas inducing T cell apoptosis	[261, 263]
		Secretion of cytokines (TGF-β and IL-10) for T cell inhibition	[264, 265]
		Release of chemokines (CCL5, CCL20, and CCL22) for Treg recruitment	[266, 267]
	Tregs	Secretion of cytokines (TGF-β and IL-10) for T cell inhibition	[271]
		IL-2 deprivation	[3]
		CD39/CD73-mediated adenosine generation	[3]
		CTLA-4 expression-mediated competition with CD28 for CD80/CD86 binding	[3]

**Table 6:** Advanced application of nanomedicines for rescuing T cell exhaustion

Strategy	Target	Mechanism	Vehicle/agents	Combination	Models	Refs.
MDSCs-targeting treatments	MDSCs	MDSC elimination	Gemcitabine-loaded nanocarriers	Anticancer adoptive T cell therapy (ACT)	EG7-OVA tumor, B16 tumor	[205, 300, 301]
		Hypoxia alleviation-mediated MDSC impediment	Platelet membrane-based co-encapsulation of metformin and IR780	PDT	4T1 tumor	[303]
		Phosphoinositide-3-kinases-γ (PI3K-γ) inhibition-mediated MDSC remodeling	IPI-549-loaded targeted polymeric nanoparticles	\	KPC98027 RFP/Luc allografts	[304]
		Disruption of MDSC expansion	Pseudoneutriphil cytokine sponges	α-PD-1	B16F10 tumor, 4T1 tumor	[305]
		MDSC modulation	RNAi and CCL2 co-loaded nanocapsules	\	In vitro MDSC	[306]
		Inhibition of MDSC recruitment	Micellar hypotoxic low molecular weight heparin-tocopherol succinate nanoparticle	Dox	B16F10 tumor lung metastases	[307]
TAMs-targeting treatments	TAMs	CCL2/CCR2 chemotaxis blockade	Monocyte-targeting cationic nanoparticles encapsulating CCR2 siRNA	\	4T1 tumor	[313]
		Reinforcing phagocytosis of TAMs	CSF1R- and SHP2-inhibitor-loaded nanoparticle	\	4T1 tumor	[315]
		Selective toxicity to TAMs	Calcium bisphosphonate nanoparticles with chelator-free radiolabeling	radioisotope therapy (RIT)	4T1 tumor	[319]
		M2-TAM repolarization	R848-loaded β-cyclodextrin nanoparticles (CDNP-R848)	anti-PD-1	MC38 tumor, B16F10 melanoma	[321]
			Liposome-encapsulated zoledronate acid treatment	\	4T1 tumor cells	[322]
			Nanoparticle encapsulating M1-like TAM-related active microRNAs	\	B16F10 tumor	[323]
			Myeloid‐derived suppressor cell (MDSC) membrane‐coated iron oxide magnetic nanoparticle	PTT, ICD	B16/F10 tumor	[327]
		Improvement of TAM targeting	Surface-mannosylated nanoparticles	aPD-L1	CT26-tumor	[329]
Tregs-targeting treatments	Tregs	Down-regulation of CTLA-4	CTLA-4-siRNA loaded PLGA nanoparticle	OVA	B16 tumor	[347]
		Preferential Treg depletion	Cyclophosphamide-loaded engineering PD-1-presenting platelets	\	postsurgery B16F10 tumor	[350]
